# Obesity enriches for tumor protective microbial metabolites and treatment refractory cells to confer therapy resistance in PDAC

**DOI:** 10.1080/19490976.2022.2096328

**Published:** 2022-07-11

**Authors:** Kousik Kesh, Roberto Mendez, Beatriz Mateo-Victoriano, Vanessa T Garrido, Brittany Durden, Vineet K Gupta, Alfredo Oliveras Reyes, Nipun Merchant, Jashodeep Datta, Santanu Banerjee, Sulagna Banerjee

**Affiliations:** a Department of Surgery, Miller School of Medicine, University of Miami, Miami, FL, USA; b Sylvester Comprehensive Cancer Center, University of Miami, Miami, FL, USA; c Miami Integrative Metabolomics Research Center, University of Miami, Miami, FL, USA

**Keywords:** Pancreatic cancer, chemoresistance, gut microbiome, obesity, queuosine S-adenosyl methionine

## Abstract

Obesity causes chronic inflammation and changes in gut microbiome. However, how this contributes to poor survival and therapy resistance in patients with pancreatic cancer remain undetermined. Our current study shows that high fat diet-fed obese pancreatic tumor bearing mice do not respond to standard of care therapy with gemcitabine and paclitaxel when compared to corresponding control diet-fed mice. C57BL6 mice were put on control and high fat diet for 1 month following with pancreatic tumors were implanted in both groups. Microbiome of lean (control) and obese (high fat diet fed) mice was analyzed. Fecal matter transplant from control mice to obese mice sensitized tumors to chemotherapy and demonstrated extensive cell death. Analysis of gut microbiome showed an enrichment of queuosine (Q) producing bacteria in obese mice and an enrichment of S-adenosyl methionine (SAM) producing bacteria in control diet-fed mice. Further, supplementation of obese animals with SAM sensitized pancreatic tumors to chemotherapy. Treatment of pancreatic cancer cells with Q increased PRDX1 involved in oxidative stress protection. In parallel, tumors in obese mice showed increase in CD133^+^ treatment refractory tumor populations compared to control animals. These observations indicated that microbial metabolite Q accumulation in high fat diet-fed mice protected tumors from chemotherapy induced oxidative stress by upregulating PRDX1. This protection could be reversed by treatment with SAM. We conclude that relative concentration of SAM and queuosine in fecal samples of pancreatic cancer patients can be developed as a potential biomarker and therapeutic target in chemotherapy refractory pancreatic cancer.

## Introduction

Over last few decades, prevalence of overweight and obesity have increased worldwide, making it one of the major risk factors for a number of cancers, including pancreatic cancer.^1–3^ Pancreatic cancer is the 3^rd^ leading cause of cancer related deaths in United States and predicted to become the 2^nd^ most common cancer by 2030. In 2020 alone, the predicted number of pancreatic cancer patients is over 55,000 with more than 90% succumbing to it. Consistent with this correlation between the rising incidence of obesity and pancreatic cancer, numerous epidemiological studies have established obesity as a risk for pancreatic cancer. The direct role of obesity in the onset, progression, and prognosis of pancreatic cancer, however, is incompletely understood. Studies have shown that the risk of pancreatic ductal adenocarcinoma (PDAC) is about 47% greater for patients with high body mass index, particularly those with increased abdominal adiposity.^4^ Studies also showed that individuals who were obese/overweight in early adulthood, had 54% increased risk for PDAC.^4^ These studies suggest that increased body weight may contribute to poor survival in PDAC patients and increased mortality by 2-fold.^5,6^ A large-scale study in 2009 showed overweight and obesity was associated with lower overall survival in patients with pancreatic cancer.^7^ However, despite these efforts, mechanisms that contribute to poor prognosis, dismal survival and resistance to therapy have remained unknown.

Diet induced obesity affects systemic parameters like inflammation, along with altering the gut microbiome significantly, thereby altering the critical balance between the host and microbial metabolites.^8^ Further, in several cancers including pancreatic cancer, gut microbiome is associated with tumor progression, response to therapy, as well as prognosis.^9–13^ In fact, tumor microbiota has also been shown to contribute to gemcitabine resistance in pancreatic cancer.^14^ While the association of microbiome in tumor progression as well as in determining its properties are being evaluated, there is a lacuna in understanding the mechanism by which bacteria or their metabolites may impact therapeutic resistance.

The role of microbial metabolites in tumor progression is one aspect of microbiome-cancer research field that has remained underexplored. While some studies show the role of short chain fatty acid (SCFA) in colon cancer progression, their role in cancers that are more remote and not exposed to the microbial milieu is not clear. Certain microbial metabolites like polyamines supplement the host metabolic pool and contribute to tumor cell proliferation by feeding into nucleotide and protein synthesis pathways.^15–17^ Among metabolites, S-adenosyl methionine or SAM has emerged as the one that regulates the balance between cell survival and cell death. Produced by bacteria as well as by mammalian cells, SAM regulates cysteine-methionine metabolism, immune response and methylation of nucleotides thus controlling transcriptional processes. SAM has been used as an anti-tumor agent as well.^18,19^ Among metabolites solely made by bacteria is a t-RNA homolog, Queuosine (Q). Q cannot be synthesized by mammalian cells; however, it is required by mammalian cells for tRNA modifications.^20–22^

Apart from altering gut microbiome, obesity also affects the therapy resistance by enriching for therapy resistant population in breast cancer^23^ and ovarian cancer^24^ . Signaling between adipokines from adipocytes and cancer cells are responsible for this enrichment. Whether similar enrichment occurs in pancreatic tumors is not known. Recently published study from our laboratory show that pro-tumorigenic cytokine IL6 can enrich for therapy resistant CD133+ population in pancreatic cancer cells.^25^ Interestingly, we as well as others show that IL6 is the major cytokine produced under obese conditions.^26,27^

In this study, we report that pancreatic tumors are resistant to the Gemcitabine/Paclitaxel chemotherapy in high fat diet-fed obese, compared with control diet-fed lean animals. Deeper analysis of the microbiome revealed an enrichment of bacteria secreting the metabolite queuosine (Q) in obese animals and enrichment of SAM secreting bacteria in lean animals. Interestingly, fecal microbial transplant (FMT) from lean to obese animals as well as supplementing resistant tumor bearing animals with SAM, sensitized high fat diet-fed murine tumors to chemotherapy, indicating a direct role of the microbial metabolites on therapy response. We further show that both high fat as well as treatment with Q upregulated PRDX1, an antioxidant protein that protects tumor cells from chemotherapy induced oxidative stress. In addition, high fat diet fed mice showed an enrichment of CD133+ treatment refractory cells that show high drug detoxification properties. Thus, the current study shows a potential dual role of obesity in inducing therapy resistance: at the systemic level, obesity induced enrichment of Q producing bacteria that is protects tumors from oxidative stress and at the microenvironmental level, obesity enriches for therapy resistant population within the tumors.

## Result

### Diet induced obesity deregulates gut microbiome in tumor bearing obese mice

**Figure 1.**
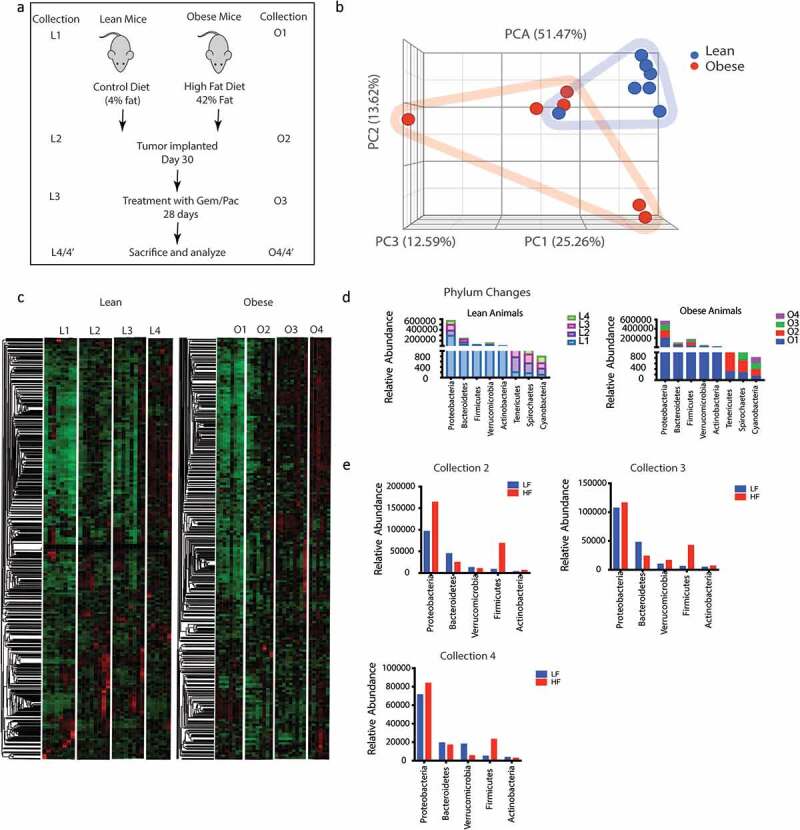
Obesity induced change in gut microbiome: Schematic diagram showing timeline of the experiment (a). High fat diet changed the gut microbiome in C57Bl6 mice (b). Differences in the microbial composition can be visualized in the heat map L1 = 1^st^ collection in lean mice before tumor implantation; L2 = 2^nd^ collection in lean mouse after 1 month of diet; L3 = 3^rd^ collection before start of Gem/Pac therapy in lean mouse, L4 = Final collection after sacrificing in lean mouse. O1 = 1^st^ collection in obese mice before tumor implantation; O2 = 2^nd^ collection in obese mouse after 1 month of diet; O3 = 3^rd^ collection before start of Gem/Pac therapy in obese mouse, O4 = Final collection after sacrificing in obese mouse (c). Dynamic changes in gut microbiome in phylum over the collection period (d,e).

To determine changes in the microbiome associated with diet-induced obesity, we analyzed the fecal microbiome in lean and obese animals after animals were placed on high fat diet and adjusted control diet, respectively, for 30 days according to the schema in Figure 1a. Collection L1-–L4 and O1–O4 represented fecal matter collection at different stages: L1/O1: Start of experiment; L2/O2: Effect of diet on microbiome; L3/O3: Effect of implanted tumor and its growth in lean and obese mice; L4/O4: End point collection of fecal matter from animals that did not receive Gem/Pac and L4’/O4’: Effect of Gem Pac therapy on microbiome change in lean and obese animals. Our data revealed differences in the microbiome composition between lean and obese mice (Figure 1b). Since each animal was tracked throughout the experiment, we next determined the changes in the composition of microbiome because of diet in a temporal fashion. Analysis of O1–O4 collection showed visible changes in the microbiome compared to L1–L4 indicating the diet induced obesity changed the microbiome both before and after tumor implantation (Figure 1c). At the phylum level, there was slightly increased abundance in Proteobacteria and Firmicutes in animals on high fat diet for 30 days (Collection O2 compared to collection L2). Similarly, we observed a decrease in the Bacteroidetes in these animals (Figure 1d). This relative abundance was maintained through collection O3 and O4 (Figure 1e). Similar relative abundance was observed at the class level as well (Supplementary Figure 1A). When we compared the gut microbiome in response to Gem/Pac treatment within each group, there was no observable difference in the relative abundance between lean and obese animals treated with Gem/Pac (Supplementary Figure 1B).

### Obesity mediated fecal microbiome confer resistance to standard of care in PDAC

**Figure 2.**
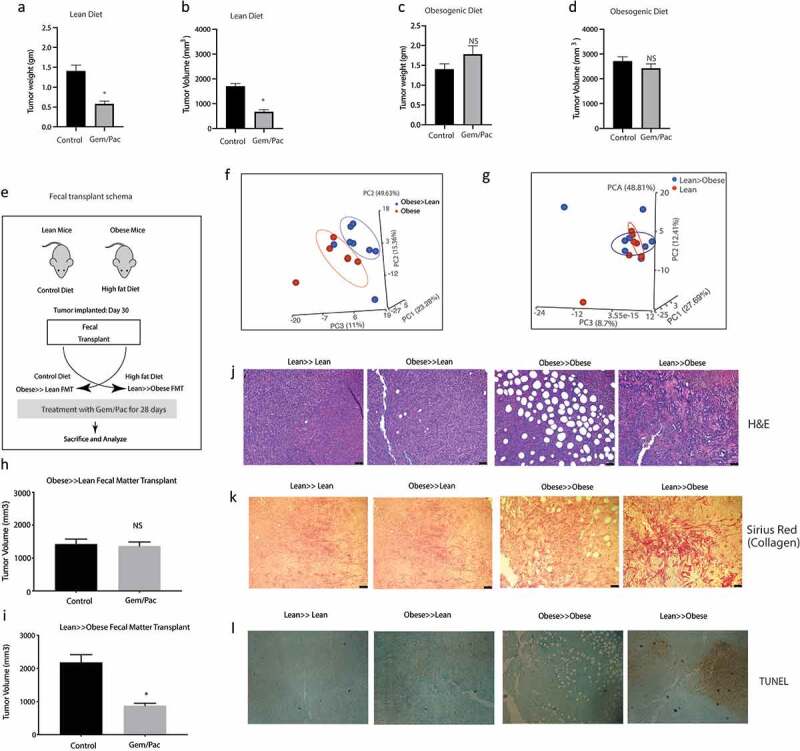
High fat diet fed mice show resistance to chemotherapy: KPC001 cells were implanted subcutaneously in C57BL6 mice on control and high fat diet and treated with Gem/Pac for 4 weeks. Tumor weight (a) and tumor volume (b) in mice on control (lean) diet showed significant decrease. Tumor weight (c) and tumor volume (d) in mice on high fat (obesogenic) diet showed no significant response. Schema for fecal transplant (e) is shown in which the high fat diet fed mice received the lean mice microbiome and vice versa. PCoA plots of Obese to lean transplant (f) and lean to obese transplant (g). Obese≫Lean FMT showed loss of response in the presence of chemotherapy (h) while Lean≫Obese FMT showed sensitivity to chemotherapy (i). Visible changes in histology was observed in the Lean≫Obese FMT in H&E slides (j), collagen deposition (k) and TUNEL staining (l).

Animals on high fat diet predictably showed increase in body weight after 30 days on their diet, whether adjusted control/lean or high fat/obesogenic (Supplementary Figure 2A). Similarly, an increase in blood glucose, triglyceride and cholesterol levels was observed in the obese mice on high fat diet compared to those on adjusted control diet (Supplementary Figure 2B–D). There was no significant difference in the tumor take between the two groups (Supplementary Figure 2E). In the animals on high fat diet, the tumor progressed faster compared to those in the adjusted control diet (Supplementary Figure 2F) and showed accumulation of lipids (Supplementary Figure 2G). Endpoint observation showed that animals in the lean group (on an adjusted control diet) responded to Gem-Pac regimen (Figure 2 a and b) while those on the high fat diet failed to respond (Figure 2 c and d). Similar observation was made when a separate mouse derived pancreatic cell line (PANC02) was used in the same experimental setting (Supplementary Figure 2H). Interestingly, upon changing the microbiome composition of the two groups of animals (lean and obese) by transplanting the fecal microbiome of lean mice to obese and vice versa right before tumor implantation (As shown in Schema Figure 2e and PCA plots Figure 2 f and g), we observed that the response of the tumors was reversed. Lean animals that previously responded to chemotherapy, stopped responding to Gem/Pac after receiving obese FMT (Figure 2h) and obese animals that we resistant to Gem/Pac started responding to this therapy after receiving the lean FMT (Figure 2i). H&E analysis of the tumor samples from the groups showed expected accumulation of infiltration of fat droplets in the tumor tissue in the obese mice compared to the lean mice. Further, obese mice receiving lean FMT showed extensive areas of necrosis in the histology (Figure 2j). Similarly, these obese mice that received lean FMT also showed widespread fibrosis and collagen deposition that was not observed in the other groups (Figure 2k). Additionally, this group of obese animals receiving lean FMT also had large areas of apoptotic cells (Figure 2l). Taken together, these data indicate that the gut microbiome may drive therapy resistance in obese PDAC-bearing animals but can be mitigated by repopulating the gut with a microbial composition derived from lean animals.

### Diet induced obesity enriches for a gut microbiome with tumor-protective microbial metabolic pathways

**Figure 3.**
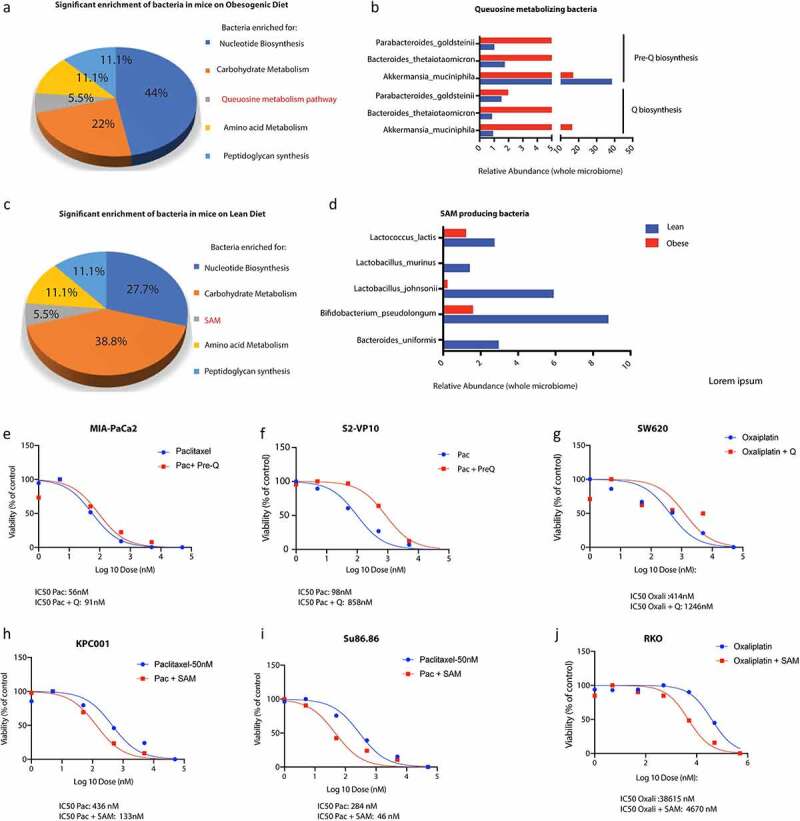
Metabolomic reconstruction using humaN2 pipeline was performed to determine the microbial metabolome. High fat diet fed mice showed an enrichment of Q metabolizing bacteria (a, b) while lean mice showed an enrichment of SAM metabolizing bacteria (c,d). Treatment of pancreatic cancer cells MIA-PACA2 (c) and S2VP10 (d) with paclitaxel and pre-Q showed a shift in IC50 indicating resistance. Treatment of colon cancer cell SW620 with oxaliplatin and pre-Q showed a similar shift in IC50 (e). Treatment of pancreatic cancer cells KPC001 (f) and Su86.86 (g) with SAM showed an opposite shift of IC50 indicating sensitization. Treatment of colon cancer cell RKO (h) with oxaliplatin and pre-Q showed a similar shift in IC50 indicating sensitization by SAM.

Since the change in gut microbiome due to high fat diet contributed to resistance to Gem/Pac therapy, we next used WGS of gut bacteria in lean and obese mice to study the enrichment of microbial populations that might contribute to this phenomenon. Our analysis revealed a distinct microbial population was enriched in the obese compared with lean mice. This included bacteria that were enriched for biosynthesis of queuosine, a tRNA homolog, contributing to protection of oncogenesis related stress (Figure 3 a and b). Similarly, analysis of the lean mice microbiome showed an enrichment of bacteria metabolizing S-adenosylmethionine or SAM (Figure 3 c and d).

To demonstrate that treatment with queuosine protects tumor cells from drug induced stress, we next treated paclitaxel sensitive MIA-PACA2 and S2VP10 cells with queuosine precursor (Pre-Q) since queuosine cannot be taken up by the cells. Our studies showed that treatment with pre-Q increased proliferation of both cancer cell lines (Supplementary Figure 3A and B) and protected them from paclitaxel induced cell death (Figure 3 e and f). To rule out disease and cell line specific effect, we treated oxaliplatin sensitive colon cancer cells with pre-Q. Similar rescue was also observed in SW620 colon cancer cells (Figure 3g).

Since our hypothesis was that microbial metabolites from lean mice sensitized the tumors to chemotherapy, we next validated if treatment with SAM sensitized normally resistant pancreatic cancer cells to Gemcitabine and Paclitaxel. We treated paclitaxel resistant SU86.86 and KPC001 cells with indicated doses of SAM along with paclitaxel and observed that SAM reversed paclitaxel resistance in these cells (Figure 3 h and i). Similar reversal of resistance was also observed in oxaliplatin resistant colon cancer cell line RKO (Figure 3j).

### Serum metabolites of obese animals showed enrichment of nitrogen metabolism and detoxification pathways

**Figure 4.**
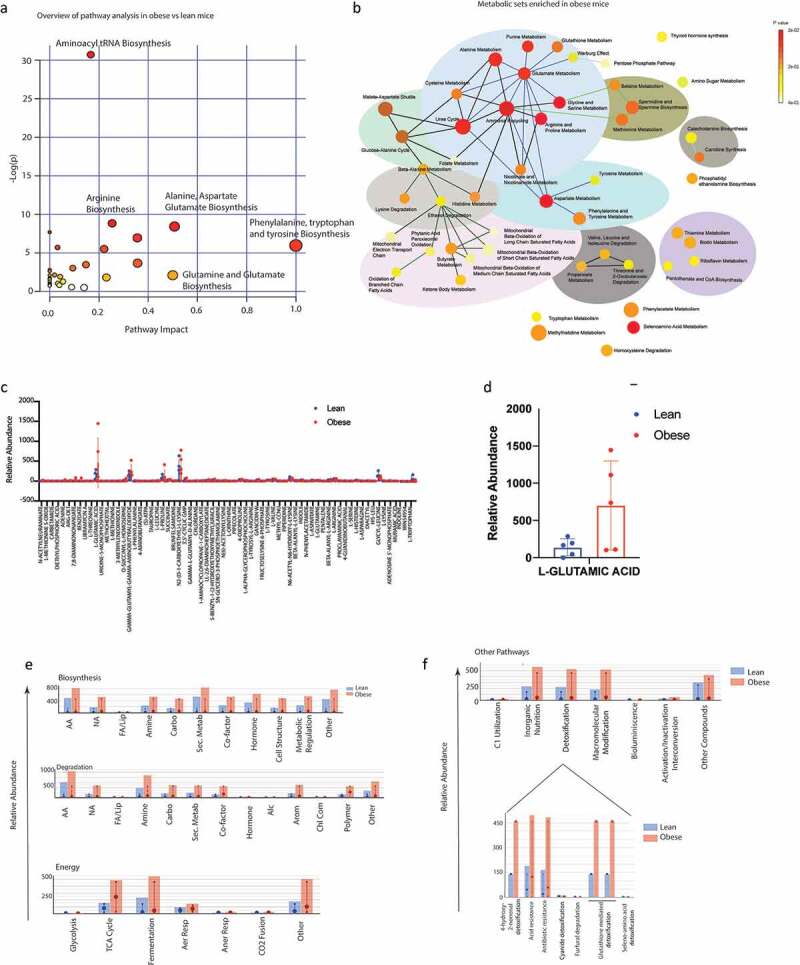
Serum metabolomics of lean and obese animals using IROA: Pathway Analysis of obese vs lean animals using MetaboAnalyst showing enrichment of critical pathways (a). MSEA of obese animals (b). Relative abundance of metabolites in lean vs obese animals (c). Obese mice showed an increase in glutamic acid (d). Metacyc showed relative changes in the metabolic pathways in lean and obese animals (e). Drug detoxification pathways were enriched in obese animals (f).

To confirm if these pathways were enriched in the serum samples as well, we next performed a Isotypic Ratio Outlier Analysis (IROA). A total of 217 different metabolites were detected in the serum of the lean and obese animals. Complete list of metabolites identified are included in Supplementary Table 1. A Metabolite Set Enrichment Analysis (MSEA) with the identified metabolites showed a significant enrichment of metabolites in aminoacyl tRNA biosynthesis followed by those involved in phenylalanine, tryptophan and tyrosine metabolism (Figure 4a). Upon separating the identified metabolites into nodes, we observed that the major node involved nitrogen metabolism encompassing urea cycle, ammonia recycling and glutamine/glutamate metabolism. Similarly, another distinct metabolic node identified was that of the mitochondrial oxidation cycle that contributes to reactive oxygen species (ROS) accumulation and beta-oxidation (Figure 4b). Upon comparing the relative abundance of the metabolites identified by IROA, L-glutamic acid/glutamine metabolism (that feeds into glutathione, proline and arginine metabolism, ammonia recycling as well as urea cycle), was found to be the most abundant metabolite in obese animals (Figure 4 c and d). An in-depth analysis of the metabolic pathways identified was next performed in MetaCyc database.^28^ We observed that there was a general upregulation of biosynthesis, degradation and energy pathways in obese animals (Figure 4e). Further, the obese animals tended to have increased accumulation of metabolites in the detoxification process, specifically glutathione (Figure 4f). This was consistent with the increased glutamic acid/glutamine accumulation in obese mice.

### S-Adenosylmethionine (SAM) is present as a fecal metabolite and sensitizes pancreatic tumor cells to chemotherapeutic agents

**Figure 5.**
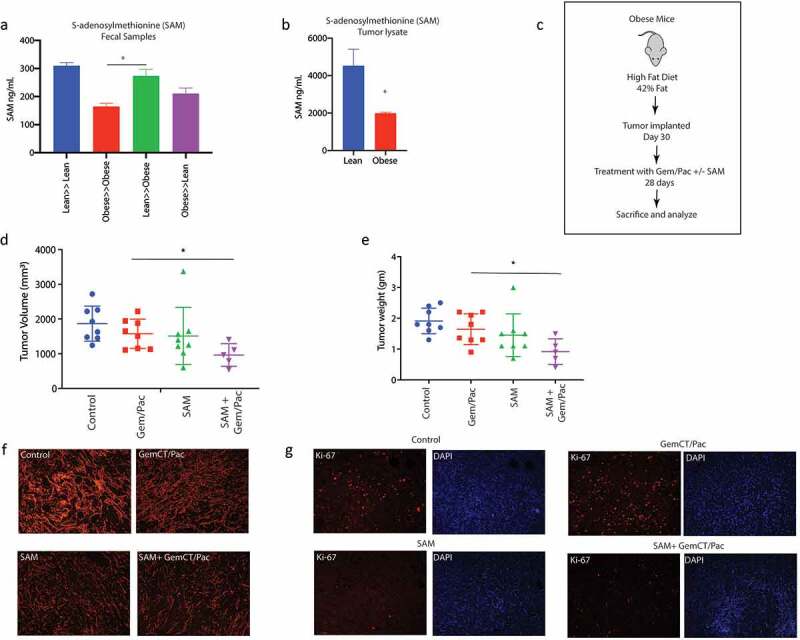
SAM reverted effect of obesity induced therapy resistance: SAM was increased in Lean≫Obese FMT animals in fecal (a) sample. SAM was decreased in Obese animals (b). Schema showing experimental set up SAM treatment in high fat diet fed, pancreatic tumor bearing mice (c). SAM decreased tumor volume (d) and weight (e). Treatment with SAM also decreased collagen (f) and Ki67+ cells (g).

Since our metabolomic reconstruction of WGS of gut bacteria in lean animals showed an enrichment of SAM metabolizing bacteria and we did not detect SAM in the serum, we next estimated SAM from the fecal samples of the lean and obese mice. Our ELISA based analysis showed that SAM was significantly elevated in the lean mice (Figure 5a). Obese mice did not have abundant SAM in their fecal samples but upon lean microbial transplant, they started producing SAM (Figure 5a). Similarly, tumors from lean mice showed increased accumulation of SAM in lean animals compared to the tumors in obese animals (Figure 5b). To study if SAM sensitized pancreatic tumors to Gem/Pac therapy in vivo, we then implanted tumors in lean and obese mice as demonstrated in Figure 5c and treated the animals with SAM in the presence of Gem/Pac standard of care. Tumor bearing obese mice that received SAM showed greater sensitivity to Gem/Pac regimen compared to those that did not receive SAM. There was significant reduction in tumor volume (Figure 5d) and weight (Figure 5e). Tissue histology showed decreased fibrosis as observed by Sirius red staining (figure 5f) and less Ki67+ cells (Figure 5g). Similar results were also observed upon implanting PANC02 cells in control and high fat fed mice (Supplementary Figure 3G).

### Queuosine induces PRDX1 expression in pancreatic cancer cells to promote resistance to chemotherapy

**Figure 6.**
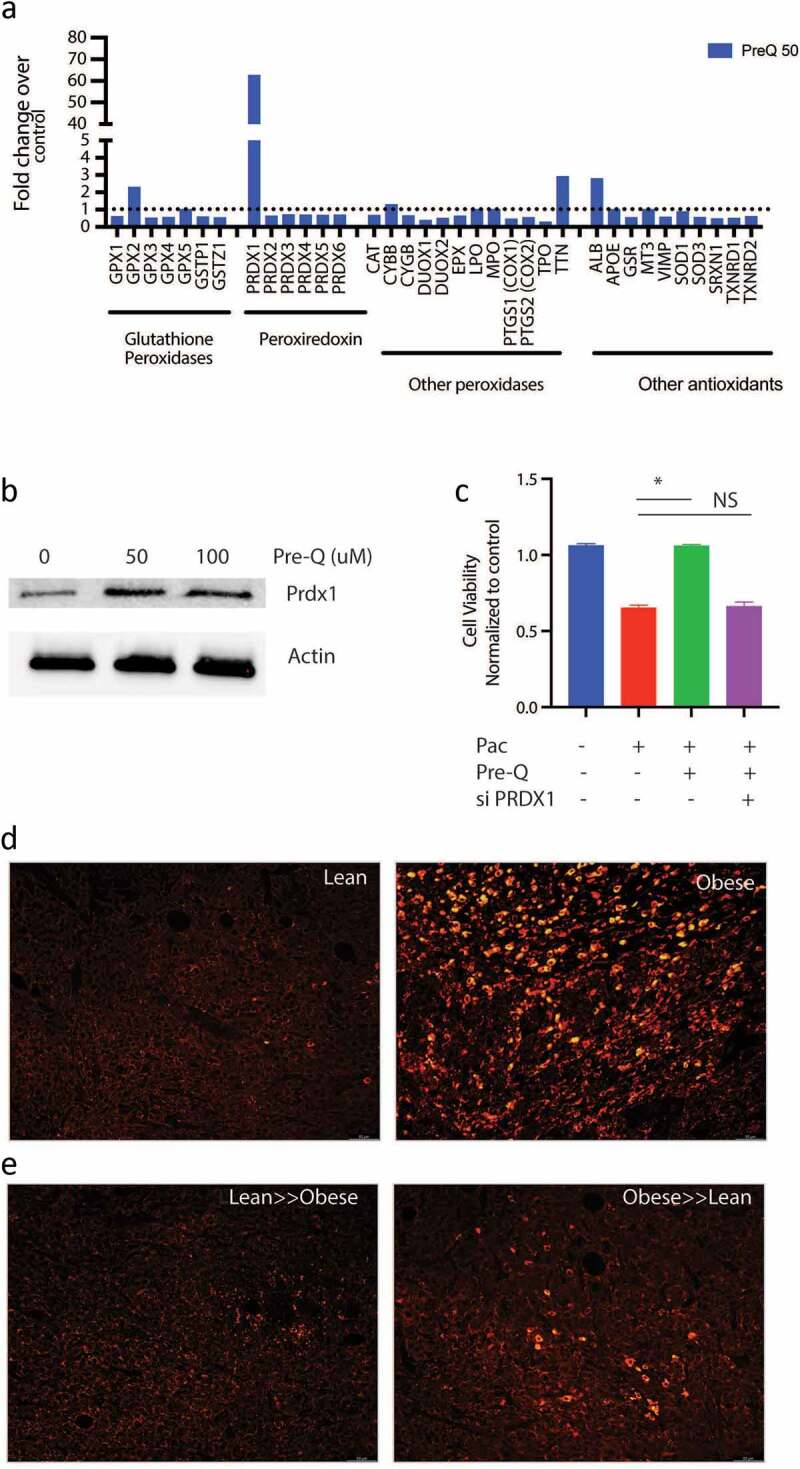
Queuosine mediated chemoresistance by upregulating PRDX1: Treatment of pancreatic cancer cell SU86.86 increased expression of PRDX1 as seen in oxidative stress PCR array analysis (a). Western blot showing upregulation of PRDX1 protein after treatment with Pre-Q (b). Silencing PRDX1 using siRNA presented Pre-Q induced resistance in MIA-PACA2 cells (c). IHC of lean and obese tumor bearing mice show upregulation of PRDX1 in obese mice (d). FMT of obese≫lean mice increased PRDX1 expression in these animals.

Chemotherapy compounds induce oxidative stress and generate ROS in cancer cells resulting in cell death. Since Q has been implicated in antioxidant defense in cells,^29^ we next studied the expression of genes involved in oxidative stress in the presence of Q using an oxidative stress PCR array. Our results showed that Q preferentially induced the expression of PRDX1 in pancreatic cancer cells SU86.86 and MIA-PACA2 (Figure 6a; Supplementary Figure 4A–C). Similar increase in Prdx1 protein expression was observed as well (Figure 6b). To study if chemoresistance in the presence of Q was via induction of PRDX1, we next inhibited PRDX1 using siRNA in pancreatic cancer cells SU86.86 and determined effect on cell viability in the presence of paclitaxel ± Q. Silencing was verified by qPCR (Supplementary Figure 4D). Silencing PRDX1 did not alter the viability of the pancreatic cancer cells (Supplementary Figure 4E). We observed that upon silencing Prdx1 in pancreatic cancer cells (MIA-PACA2), queuosine was unable to protect the cells from paclitaxel induced cell death (Figure 6c).

To confirm that in high fat fed mice had increased PRDX1 expression, we performed immunofluorescence on the tumors. Tumor bearing lean mice had low expression of Prdx1, while the high fat diet fed obese mice had a high expression of Prdx1 (Figure 6d). Upon fecal matter transplant from lean to obese decreased Prdx1 expression in obese mice while obese to lean fecal transplant increased the expression of this protein (Figure 6e), consistent with the chemoresistance observed in Figure 1.

### Obesity enriches for intra-tumoral treatment refractory population in pancreatic cancer

**Figure 7.**
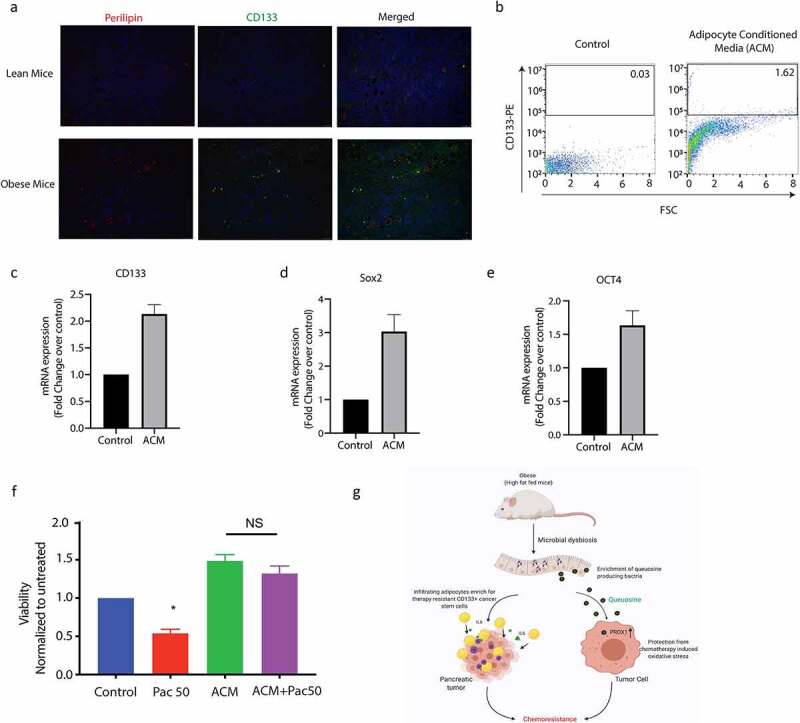
Obesity enriched for resistant CD133+ cells in pancreatic tumors. High fat diet fed tumor bearing mice showed an increase in CD133+ cells near lipid droplets (a). CD133+ population was increased when pancreatic cancer cells were treated with adipocyte conditioned media (b). Adipocyte conditioned media also increased expression of self-renewal genes like CD133 (c), Sox2 (d), Oct4 (e) and induced resistance to paclitaxel (f). Schematic diagram showing mechanism of obesity induced resistance.

Obesity and adipocyte mediated signaling affects intra-tumoral oncogenic signaling along with causing system-wide inflammatory responses.^30^ Complete analysis of the serum cytokines using multiplexed assays showed increased IL6 among the elevated cytokines in the obese mice (Supplementary Figure 5A). Interestingly, our recently published research^25^ showed that presence of IL6 enriched for a CD133+ treatment refractory population of cells in pancreatic cancer. Previous studies from our laboratory show that resistance to therapy in pancreatic tumors correlated with presence of CD133+ cells.^31,32^ Thus to study if obesity induced peritumoral adipocytes resulted in enrichment of CD133+ population in the tumor tissues, we next evaluated CD133+ cells in the tumors from obese mice. Our results showed that CD133+ cells were increased in these animals (compared to the lean animals) and tended to accumulate around the perilipin stained adipocytes in our animal models (Figure 7a, Supplementary Figure 5B). To study if adipocytes enriched for CD133+ therapy resistant population in pancreatic tumors, we next treated MIA-PACA2 (pancreatic cancer cells with negligible CD133+ population) with conditioned media from patient derived adipocytes. Our studies showed that treatment with adipocyte conditioned media made MIA-PACA2 cells resistant to paclitaxel (Figure 7b). Further analysis showed that this treatment also led to a distinct enrichment of CD133+ population (Figure 7c, Supplementary Figure 5C). Further, expression of several drug resistance genes was elevated when pancreatic cancer cells were treated with adipocyte conditioned media or with pre-Q (Supplementary Figure 5D). Additionally, adipocyte conditioned media also enriched for stemness genes in the MIA-PACA2 cells (Figure 7d–f) Analysis of the adipocyte conditioned media also revealed IL6 as the major cytokine produced by the cells (Supplementary Figure 5E). To study if queuosine increased stemness to promote chemoresistance, we next treated pancreatic cancer cells MIA-PACA2 and Su86.86 with pre-Q. We observed no change in the mRNA expression of stemness or self-renewal genes (Supplementary Figure 5F).

## Discussion

Research on effect of obesity on different cancers has been gaining importance ever since onset of obesity at early years and sedentary lifestyle has become predominant. While systemic factors like chronic inflammation, hormones, circulating adipokines, and adipocyte-mediated inflammatory and immunosuppressive microenvironment and gut microbial dysbiosis have been attributed to the association of obesity with pancreatic cancer,^1,2,8^ adipocyte mediated intratumor processes have also been ascribed to poor prognosis of the disease. Over the past decade, growing evidence has shown that the composition of the gut microbiota and its activity might be associated not only with the onset of inflammation but also with metabolic disorders and cancer.

In accordance with this, studies from our laboratory have shown that gut microbiome is associated with pancreatic cancer progression.^9^ Microbial dysbiosis has been correlated with therapy response in a number of cancers.^10–12^ Our ongoing and recently published studies also indicated that conditions of high blood glucose (as seen in Type 2 diabetes) also contribute to chemoresistance in pancreatic cancer pre-clinical models.^13^ Similarly, tumor microbiota has been shown to contribute to gemcitabine resistance in pancreatic cancer.^14^ While the association of microbiome in tumor progression as well as in determining its properties are being evaluated, there is a lacuna in understanding the mechanism by which bacteria may affect these processes. While this was observed using two different primary mouse cell lines, detailed study using genetically engineered mouse models for different cancers are needed to ascertain if mechanism of obesity driven therapy resistance is being mediated by gut bacterial metabolites in other cancers as well.

In the gut, the microbiome, its metabolites are in a constant state of flux with the host tissue and its secretome. Several microbial metabolites like SCFAs, trimethylamine and polyamines have been directly implicated in driving tumorigenesis by contributing to protein and nucleotide synthesis for the rapidly proliferating tumor cells. Several studies show an enrichment of bacterial species that metabolize antioxidants and thus contribute to therapy resistance in multiple cancers.^13,33,34^ In this study, we observed that in obese animals, pancreatic tumors did not respond to standard of care treatments like Gemcitabine/Paclitaxel cocktail. Interestingly, when the microbial composition of the lean and the obese animals was changed by FMT, the obese mice started responding to therapy. A deeper analysis of the microbiome revealed an enrichment of bacteria secreting the bacterial metabolite queuosine (Q) in the obese animals and an enrichment of SAM secreting bacteria in the lean animals (Figure 3). Originally identified in E. coli, queuosine, Q was found to occupy the first anticodon position of tRNAs for histidine, aspartic acid, asparagine and tyrosine.^35^ The hyper-modified nucleobase of queuosine is queuine. In mammalian cells, queuine treatment is reported to modulate tolerance to hypoxia,^36^ influence proliferation^37,38^ and the expression of lactate dehydrogenase^39^ . Interestingly, we observe that bacterial taxa A. muciniphilia to be slightly enriched in pre-Q biosynthesis pathway in lean mice as well as in queuosine biosynthesis in obese mice (Figure 3b). Pre-Q can be utilized for synthesis of archeosine, tocoyamycin as well as queuosine in bacteria. It is possible that in lean mice, Pre-Q is not completely metabolized to Q, resulting in their sensitivity to the chemotherapeutic compounds.

Q is a tRNA homolog, that protects from oncogenesis induced stress during tumor progression. Queuosine is also a modified nucleoside, the occurrence of which is widespread across the animal and plant kingdoms. Yet eukaryotes are unable to synthesize Q-nucleoside or any of its precursor forms. Instead, they salvage the nucleobase of queuosine, referred to as queuine or Q-base. In the case of metazoans, the source of queuine is dietary, whether from the gut microflora or from ingested food.^40^ In HeLa cells cultured in medium containing 10% horse serum, queuine treatment increased cell density under aerobic conditions but decreased cell density under hypoxic conditions,^41^ suggesting that that queuine is a stimulant for proliferation in an aerobic environment, but inhibitory when conditions are hypoxic. Later, a study from the same group on the proliferation of non-transformed, transformed and tumor-derived cell lines concluded that queuine can stimulate or inhibit growth, depending on the cell line investigated.^38^ Recent studies have shown the queuine can promote anti-oxidant defense system by activating cellular antioxidant enzyme activity in cancer.^29^ It is well known that obesity mediated inflammation leads to generation of ROS in the cells. Interestingly, a study published in 2012 reported the structural organization of the enzyme GluQ-RS in bacteria, that was responsible for formation of GluQ tRNA modification.^42^ This study and two others also showed that this enzyme required a high concentration of glutamate to be activated in the host so that it could be transferred to the queuosine base present on the tRNA.^Asp43,44^ Thus, enrichment of queuosine metabolizing bacteria as well as accumulation of metabolites that promoted detoxification of xenobiotics in the tumor-bearing obese mice was indicative of protection from drug induced stress. Our study showed that treatment with pre-Q upregulated expression of PRDX1 in cells (Figure 6 a and b) and obesity triggered its upregulation in tumor bearing animals (Figure 6d). Also, since silencing PRDX1 sensitized the pancreatic cancer cells to paclitaxel even in the presence of Pre-Q indicated that Q induced chemoresistance was being mediated via PRDX1 (Figure 6c).

SAM is known to be an anti-tumor metabolite. In gastric and colon cancer, SAM reverses hypomethylation status of c-myc and H-ras to inhibit tumor growth^45^ . Similarly, in breast cancer, treatment with SAM and doxorubicin showed anti-proliferative as well enhanced apoptotic properties.^46^ We detected SAM in the fecal as well as tumor samples of the tumor bearing lean animals. Based on this observation, we hypothesized that microbial metabolite Q accumulates in the obese animals and offers them protection from oxidative stress associated with chemotherapy. Upon transplanting the microbiome of the obese animals with that of the lean animals, this protection is lost, and the tumors start responding to chemotherapy. Similarly, accumulation of SAM in lean animals sensitizes the tumors in them to chemotherapy. When replaced with the obese microbiome, this sensitization is lost, and tumors stop responding to chemotherapy. In this study we have validated this hypothesis and observed that treatment with pre-queuosine offers therapy resistance to pancreatic cancer cells, while treatment with SAM sensitizes them both in vitro and in vivo.

Cancer stem cells are known to be enriched under conditions of obesity. Our data corroborated that (Figure 7). Adipocyte conditioned media showed enrichment in CD133+ population as well as increased expression of stemness genes. Interestingly, treatment with Q did not seem to affect the cancer stemness (Supplementary Figure 5E). This indicated that obesity induced poor prognosis and therapy resistance was being mediated both by enrichment of cancer stem cells from the pro-inflammatory cytokines secreted by the accumulated adipocytes in the microenvironment as well as by the microbial metabolite Q. This mechanism of resistance is summarized in Figure 7g.

## Conclusion

This study shows for the first time that microbial metabolites like Queuosine contribute to therapy resistance in pancreatic cancer under conditions of obesity by upregulating PRDX1, which protects them from chemotherapy induced oxidative stress. We further show that this therapy resistance can be reversed by FMT from lean mice as well as by SAM, another metabolite produced by gut bacteria. This finding can be of potential significance in case of pancreatic cancer patients that do not respond to standard of care as these “non-responding” patients can be made to “respond” to therapy by supplementing with S-adenosine methionine or SAM. This has immense potential in improving the survival statistics of patients diagnosed with pancreatic cancer.

## Material and method

### Experimental animals

C57BL/6 J mice were obtained from the Jackson Laboratory (Bar Harbor, Maine, USA). All mice were male and 4–6 weeks old. Food and tap water were available ad libitum. All mice were housed four mice per cages and maintained on a 12-h light/dark cycle, in a constant temperature (72 ± 1 °F) and 50% humidity. All procedures were conducted according to the protocols approved by the University of Miami Institutional Animal Care and Use Committee (IACUC).

### Animal model for diet induced obesity

C57BL/6 J mice, both male and female were first randomly divided into two groups [Obese group and Lean group]. Mice in the Obese group were given a high caloric diet brand name TD.88137, adjusted calories diet (42% from fat) and Lean group feed adjusted control diet named TD.08405 adjusted control diet (4% from fat) (Envigo, USA) during the total experiment period. After 4 weeks on designated diet, the weight gain was monitored for both groups. Additionally, blood glucose levels, serum triglyceride levels and blood cholesterol levels were measured to validate establishment of model. Calory intake was monitored by measuring the chow on a daily basis.

### Blood glucose, triglyceride and cholesterol measurement

Blood samples were collected by retro-orbital sinus puncture via the medial canthus of the eye using clean 44.7-μ L heparinized micro hematocrit tubes. No anesthesia was used at the time of the blood sampling, to avoid unequal variations between animals and avoid the effects of anesthesia on the blood glucose levels. Mice blood glucose was measured using true track blood glucose meter and strips (Trivida health). Measurement of total cholesterol and triglyceride from mice serum were performed using cholesterol assay kit (Abcam) and triglyceride assay kit (Sigma-Aldrich) according to the manufacturer’s instructions.

### Tumor implantation

5 × 10^3^ number of pancreatic cancer cells (KPC001, primary mouse pancreatic tumor cells) and 5 × 10^4^ PANC02 (primary mouse pancreatic cancer cells) were implanted in both groups of mice after 4 weeks of diet. After 2 weeks of tumor implantation when subcutaneous pancreatic cancer model was established, the two groups of mice were each further randomly divided into two subgroups (Obese and Obese + Gem/Pac; Lean and Lean +Gem/Pac). The Gem/Pac groups in both obese and lean mice received intraperitoneal injections of 100 mg/kg of gemcitabine and 10 mg/kg of paclitaxel twice in a week for consecutive 4 weeks, while the HF and LF groups were only receive equal volume of saline.

In another set of experiment 32 mice were similarly divided in to two groups which further subdivided in four groups (Obese, Lean, Obese+Gem/Pac, Lean+ Gem/Pac) after 1 month feeding. These mice served as tumor free control and sacrifice at similar time point.

### Reciprocal Fecal microbial transplantation experiment

Forty-eight mice were divided in to two groups named Obese (donor) and Lean (donor) and received High Fat and Adjusted Control diet respectively. These animals serve as a fecal microbial donor pool. After one month of feeding animals were sacrificed and stool sample were collected aseptically. Stool were preserved at −80C for future used. 200 mg of the fecal extract was suspended in 1 mL sterile PBS, filtered through 70-µm cell strainer, and centrifuged at 6000 ×g for 20 min. About 10^10^ CFU/mL fecal bacteria were suspended in 6% NaHCO_3_ buffer with 20% sucrose. Thirty-two mice were divided into Obese and Lean group (16 mice each) and feed for 1 month with High Fat and Adjusted Control diet. Obese mice were further randomized into Obese, Lean≫Obese (FMT) and Lean mice divided into Lean, Obese≫Lean (FMT). Lean≫Obese (FMT) mice were continuously feed in HF diet but received oral gavage of Lean mice stool for three times in a week for continuous 6 weeks similarly Obese≫Lean (FMT) received HF stool remain in adjusted control diet.

### In vivo treatment of SAM

Sixty-four mice were divided equally and feed with high fat and adjusted control diet separately for 1 month. HF group further dived in to four groups as Obese, Obese+GP, Obese+SAM, Obese+GP+SAM. Lean groups also divided similarly. Subcutaneous pancreatic tumor cells (KPC001 and PANC02) were implanted in each groups at day 30. From day 45 onwards treatment were started with either Gem/Pac alone or with SAM (Sigma Aldrich) or SAM alone. SAM were dissolve in saline and given 100 mg/kg BW every day for 4 weeks. Mice were sacrificed. Tumor volume and weight were measured.

### Fecal matter collection and DNA isolation

Fecal samples were collected in different time point to understand the effect of several sequential treatment in gut microbiota. Fecal samples collection was performed at day 1, 45, 60 and 90. Samples were collected in a sterile Eppendorf tube inside a biosafety cabinet with sterile forceps. Each group consisting of eight animals were randomized (group wise) to nullify cage-effect in microbiome studies among the groups. After 90 days, all animals were sacrificed according to protocols approved by University of Miami Animal Care Committee. Part of the tumor sample were flash frozen in liquid nitrogen, while the rest were formalin fixed for paraffin embedding and histochemical analysis. Blood was collected by cardiac puncture prior to euthanizing the animals. Serum samples were stored for biochemical analysis. DNA from the murine fecal samples was isolated using the Power Soil DNA Isolation Kit (Qiagen) according to manufacturer’s instructions. All samples were quantified using the Qubit® Quant-iT dsDNA High-Sensitivity Kit (Invitrogen, Life Technologies, Grand Island, NY) to ensure that they met minimum concentration and mass of DNA and were submitted to University of Minnesota Genomics Center for Whole Genome Sequencing.

### Metagenomic sequencing and microbiome analysis

Shotgun metagenomic library was constructed from fecal DNA with the Nextera DNA sample preparation kit (Illumina, San Diego, CA), as per manufacturer’s specification. Barcoding indices were inserted using Nextera indexing kit (Illumina). Products were purified using Agencourt AMpure XP kit (Beckman Coulter, Brea, CA) and pooled for sequencing. Samples were sequenced using MiSeq reagent kit V2 (Illumina) in a HiSeq2500 sequencer.

Raw sequences were sorted using assigned barcodes and cleaned up before analysis (barcodes removed and sequences above a quality score, Q ≥ 30 taken forward for analyses). For assembly and annotation of sequences, MetAMOS^47^ pipeline or Partek Flow software (Partek® Flow®, Partek Inc., St. Louis, MO) were used. These softwares provide powerful tools to filter unique hits between human and mouse-specific genes versus microbial signatures. Alpha and Beta diversity calculations were done using embedded programs within the metagenomic pipeline, or using Stata15 (StataCorp LLC, College Station, TX) or EXPLICET software.^48^ Functional profiling was performed using HUMAnN2-0.11.1^49^ with Uniref50 database to implement KEGG orthologies.

### Histology and and TUNEL assay

Tumor from all groups of mice were sectioned for histological studies. The tissue samples were fixed in 10% formalin and embedded in paraffin. The sections (5 μm) were cut using microtome, stained with hematoxylin and eosin, and slides were assessed using microscope (Leica microsystems, Germany) using at original magnification 10× and processed in Adobe Photoshop. For TUNEL study, paraffin embedded sections were deparaffinized with xylene followed by rehydration with descending alcohol series. Study was performed according to the manufacturer protocol (Abcam).

### Sirius red staining and measurements

Tissue sections were deparaffinized and hydrated in a descending order of alcohol solution, followed by PBS washing. Collagen staining were performed using picrosirius red staining solution (Chondrex Inc). The sections were washed with acidified water and dehydrated using absolute alcohol followed by mount in a resinous medium. The Sirius red–stained area was quantified using ImageJ software by selecting stained fibers in randomly selected five fields at a magnification of 10× under a light microscope.

### Cell culture

Pancreatic cancer cell line MIAPaCa-2 and SU86.86 was purchased from ATCC, KPC001, KPC23 was isolated from the KRAS^G12D^ TP53^R172H^Pdx-Cre spontaneous mouse model from pancreatic cancer; S2VP10 was a gift from Masato Yamamoto (University of Minnesota, MN); colon cancer cell line (SW620 and RKO) were a gift from David Robbins (U Miami). Pan02 cells were obtained as a gift from Dr. Merchant, University of Miami. S2VP10 was cultured in RPMI supplemented with 10% fetal bovine serum (FBS) with 1% Pen Strep (Life Technologies). All others were cultured in DMEM high glucose (Hyclone) containing 10% FBS with 1% Pen Strep (Gibco). All the established cell lines were used between passages 5 and 18. All cells were maintained at 37°C in a humidified air atmosphere with 5% CO_2_. 70% confluent cells were used in each experiment. Cell lines were routinely checked for mycoplasma contamination and verified by STR profiling.

Small molecules inhibitor pre-queuosine 1 (50 uM, Sigma Aldrich), SAM (200 uM, Sigma) and paclitaxel (50 nM, Sigma Aldrich) were used in various experiment.

### Isotope Ratio Outlier Analysis (IROA)

IROA was done on serum samples from animals on high fat and control diet following protocols described in^50^and analyzed in Q-Exactive mass spectrometer.

### Statistical analysis

Data were presented as the Mean ± SEM. Statistical analyses were performed using GraphPad Prism software, version 8.0. Differences between two groups were analyzed by Student’s *t* test. *P* < .05 was considered statistically significant. Most statistical functions for microbiome and metabolome were embedded within MetAMOS^47^ pipeline or Partek Flow software (Partek® Flow®, Partek Inc., St. Louis, MO). Output files from microbial sequence analysis and predictive metabolomics were further subjected to groupwise comparison. Depending on the analysis (as mentioned in respective figure legends), test of significance was either Mann–Whitney U test (Graphpad Prism), one-way ANOVA or two-tailed t-test with false discovery rate (FDR) correction (using Bonferroni or Benjamini-Hochberg correction). The FDR threshold was set at 0.1 and *p* < .05 was considered to be significant.

## Supplementary Material

Supplemental Material

## Data Availability

All data generated or analyzed during this study are included in this published article [and its supplementary information files].

## References

[1] Pothuraju R, Rachagani S, Junker WM, Chaudhary S, Saraswathi V, Kaur S, Batra SK. Pancreatic cancer associated with obesity and diabetes: an alternative approach for its targeting. J Exp Clin Cancer Res. 2018;37:319. doi:10.1186/s13046-018-0963-4. 30567565 PMC6299603

[2] Cascetta P, Cavaliere A, Piro G, Torroni L, Santoro R, Tortora G, Melisi D, Carbone C. Pancreatic cancer and obesity: molecular mechanisms of cell transformation and chemoresistance. Int J Mol Sci. 2018;19:3331. doi:10.3390/ijms19113331. PMC6274743 30366466

[3] Rawla P, Thandra KC, Sunkara T. Pancreatic cancer and obesity: epidemiology, mechanism, and preventive strategies. Clin J Gastroenterol. 2019;12:285–18. doi:10.1007/s12328-019-00953-3. 30788774

[4] Genkinger JM, Spiegelman D, Anderson KE, Bernstein L, van den Brandt PA, Calle EE, English DR, Folsom AR, Freudenheim JL, Fuchs CS, et al. A pooled analysis of 14 cohort studies of anthropometric factors and pancreatic cancer risk. Int J Cancer. 2011;129:1708–1717. doi:10.1002/ijc.25794. 21105029 PMC3073156

[5] McWilliams RR, Matsumoto ME, Burch PA, Kim GP, Halfdanarson TR, de Andrade M, Reid-Lombardo K, Bamlet WR. Obesity adversely affects survival in pancreatic cancer patients. Cancer. 2010;116:5054–5062. doi:10.1002/cncr.25465. 20665496 PMC2963722

[6] Calle EE, Rodriguez C, Walker-Thurmond K, Thun MJ. Overweight, obesity, and mortality from cancer in a prospectively studied cohort of U.S. adults. N Engl J Med. 2003;348:1625–1638. doi:10.1056/NEJMoa021423. 12711737

[7] Li D, Morris JS, Liu J, Hassan MM, Day RS, Bondy ML, Abbruzzese JL. Body mass index and risk, age of onset, and survival in patients with pancreatic cancer. JAMA. 2009;301:2553–2562. doi:10.1001/jama.2009.886. 19549972 PMC2760963

[8] Cani PD, Jordan BF. Gut microbiota-mediated inflammation in obesity: a link with gastrointestinal cancer. Nat Rev Gastroenterol Hepatol. 2018;15:671–682. doi:10.1038/s41575-018-0025-6. 29844585

[9] Mendez R, Kesh K, Arora N, Di Martino L, McAllister F, Merchant N, Banerjee S, Banerjee S. Microbial dysbiosis and polyamine metabolism as predictive markers for early detection of pancreatic cancer. Carcinogenesis. 2020;41:561–570. doi:10.1093/carcin/bgz116. 31369062 PMC7350554

[10] Zheng Y, Wang T, Tu X, Huang Y, Zhang H, Tan D, Jiang W, Cai S, Zhao P, Song R, et al. Gut microbiome affects the response to anti-PD-1 immunotherapy in patients with hepatocellular carcinoma. J Immunother Cancer. 2019;7:193. doi:10.1186/s40425-019-0650-9. 31337439 PMC6651993

[11] Routy B, Le Chatelier E, Derosa L, Duong CPM, Alou MT, Daillere R, Fluckiger A, Messaoudene M, Rauber C, Roberti MP, et al. Gut microbiome influences efficacy of PD-1-based immunotherapy against epithelial tumors. Science. 2018;359:91–97. doi:10.1126/science.aan3706. 29097494

[12] Mima K, Nakagawa S, Sawayama H, Ishimoto T, Imai K, Iwatsuki M, Hashimoto D, Baba Y, Yamashita Y-I, Yoshida N, et al. The microbiome and hepatobiliary-pancreatic cancers. Cancer Lett. 2017;402:9–15. doi:10.1016/j.canlet.2017.05.001. 28527946

[13] Kesh K, Mendez R, Abdelrahman L, Banerjee S, Banerjee S. Type 2 diabetes induced microbiome dysbiosis is associated with therapy resistance in pancreatic adenocarcinoma. Microb Cell Fact. 2020;19:75. doi:10.1186/s12934-020-01330-3. 32204699 PMC7092523

[14] Geller LT, Barzily-Rokni M, Danino T, Jonas OH, Shental N, Nejman D, Gavert N, Zwang Y, Cooper ZA, Shee K, et al. Potential role of intratumor bacteria in mediating tumor resistance to the chemotherapeutic drug gemcitabine. Science. 2017;357:1156–1160. doi:10.1126/science.aah5043. 28912244 PMC5727343

[15] Tsvetikova SA, Koshel EI. Microbiota and cancer: host cellular mechanisms activated by gut microbial metabolites. Int J Med Microbiol. 2020;310:151425. doi:10.1016/j.ijmm.2020.151425. 32423739

[16] Cueva C, Silva M, Pinillos I, Bartolome B, Moreno-Arribas MV. Interplay between dietary polyphenols and oral and gut microbiota in the development of colorectal cancer. Nutrients. 2020;12:625. doi:10.3390/nu12030625. PMC7146370 32120799

[17] Elinav E, Garrett WS, Trinchieri G, Wargo J. The cancer microbiome. Nat Rev Cancer. 2019;19:371–376. doi:10.1038/s41568-019-0155-3. 31186547 PMC6700740

[18] Mehdi A, Attias M, Mahmood N, Arakelian A, Mihalcioiu C, Piccirillo CA, Szyf M, Rabbani SA. Enhanced anticancer effect of a combination of S-adenosylmethionine (SAM) and immune checkpoint inhibitor (ICPi) in a syngeneic mouse model of advanced melanoma. Front Oncol. 2020;10:1361. doi:10.3389/fonc.2020.01361. 32983966 PMC7492272

[19] Mahmood N, Arakelian A, Cheishvili D, Szyf M, Rabbani SA. S-adenosylmethionine in combination with decitabine shows enhanced anti-cancer effects in repressing breast cancer growth and metastasis. J Cell Mol Med. 2020;24:10322–10337. doi:10.1111/jcmm.15642. 32720467 PMC7521255

[20] Morris RC, Elliott MS. Queuosine modification of tRNA: a case for convergent evolution. Mol Genet Metab. 2001;74:147–159. doi:10.1006/mgme.2001.3216. 11592812

[21] Slany RK, Kersten H. Genes, enzymes and coenzymes of queuosine biosynthesis in procaryotes. Biochimie. 1994;76:1178–1182. doi:10.1016/0300-9084(94)90047-7. 7748953

[22] Tuorto F, Legrand C, Cirzi C, Federico G, Liebers R, Muller M, Ehrenhofer‐Murray AE, Dittmar G, Gröne H-J, Lyko F, et al. Queuosine-modified tRNAs confer nutritional control of protein translation. EMBO J. 2018;37. doi:10.15252/embj.201899777. PMC6138434 30093495

[23] Bao B, Teslow EA, Mitrea C, Boerner JL, Dyson G, Bollig-Fischer A. Role of TET1 and 5hmC in an obesity-linked pathway driving cancer stem cells in triple-negative breast cancer. Mol Cancer Res. 2020;18:1803–1814. doi:10.1158/1541-7786.MCR-20-0359. 32913111 PMC7718329

[24] Huang H, Wang Y, Kandpal M, Zhao G, Cardenas H, Ji Y, Chaparala A, Tanner EJ, Chen J, Davuluri RV, et al. FTO-dependent N 6 -methyladenosine modifications inhibit ovarian cancer stem cell self-renewal by blocking cAMP signaling. Cancer Res. 2020;80:3200–3214. doi:10.1158/0008-5472.CAN-19-4044. 32606006 PMC7442742

[25] Kesh K, Garrido VT, Dosch A, Durden B, Gupta VK, Sharma NS, Lyle M, Nagathihalli N, Merchant N, Saluja A, et al. Stroma secreted IL6 selects for “stem-like” population and alters pancreatic tumor microenvironment by reprogramming metabolic pathways. Cell Death Dis. 2020;11:967. doi:10.1038/s41419-020-03168-4. 33177492 PMC7658205

[26] Hayashi T, Fujita K, Nojima S, Hayashi Y, Nakano K, Ishizuya Y, Wang C, Yamamoto Y, Kinouchi T, Matsuzaki K, et al. High-fat diet-induced inflammation accelerates prostate cancer growth via IL6 signaling. Clin Cancer Res. 2018;24:4309–4318. doi:10.1158/1078-0432.CCR-18-0106. 29776955

[27] Wunderlich CM, Ackermann PJ, Ostermann AL, Adams-Quack P, Vogt MC, Tran ML, Nikolajev A, Waisman A, Garbers C, Theurich S, et al. Obesity exacerbates colitis-associated cancer via IL-6-regulated macrophage polarisation and CCL-20/CCR-6-mediated lymphocyte recruitment. Nat Commun. 2018;9:1646. doi:10.1038/s41467-018-03773-0. 29695802 PMC5916940

[28] Caspi R, Billington R, Keseler IM, Kothari A, Krummenacker M, Midford PE, Ong WK, Paley S, Subhraveti P, Karp PD, et al. The MetaCyc database of metabolic pathways and enzymes - a 2019 update. Nucleic Acids Res. 2020;48:D445–D53. doi:10.1093/nar/gkz862. 31586394 PMC6943030

[29] Pathak C, Jaiswal YK, Vinayak M. Queuine promotes antioxidant defence system by activating cellular antioxidant enzyme activities in cancer. Biosci Rep. 2008;28:73–81. doi:10.1042/BSR20070011. 18290765

[30] Iyengar NM, Gucalp A, Dannenberg AJ, Hudis CA. Obesity and cancer mechanisms: tumor microenvironment and inflammation. J Clin Oncol. 2016;34:4270–4276. doi:10.1200/JCO.2016.67.4283. 27903155 PMC5562428

[31] Gupta VK, Sharma NS, Kesh K, Dauer P, Nomura A, Giri B, Dudeja V, Banerjee S, Bhattacharya S, Saluja A, et al. Metastasis and chemoresistance in CD133 expressing pancreatic cancer cells are dependent on their lipid raft integrity. Cancer Lett. 2018;439:101–112. doi:10.1016/j.canlet.2018.09.028. 30290209

[32] Nomura A, Dauer P, Gupta V, McGinn O, Arora N, Majumdar K, Iii CU, Dalluge J, Dudeja V, Saluja A, et al. Microenvironment mediated alterations to metabolic pathways confer increased chemo-resistance in CD133+ tumor initiating cells. Oncotarget. 2016;7:56324–56337. doi:10.18632/oncotarget.10838. 27472388 PMC5302917

[33] Bae S, Ulrich CM, Neuhouser ML, Malysheva O, Bailey LB, Xiao L, Brown EC, Cushing-Haugen KL, Zheng Y, Cheng TYD, et al. Plasma choline metabolites and colorectal cancer risk in the Women’s Health Initiative Observational Study. Cancer Res. 2014;74:7442–7452. doi:10.1158/0008-5472.CAN-14-1835. 25336191 PMC4268282

[34] Oellgaard J, Winther SA, Hansen TS, Rossing P, von Scholten BJ. Trimethylamine N-oxide (TMAO) as a new potential therapeutic target for insulin resistance and cancer. Curr Pharm Des. 2017;23:3699–3712. doi:10.2174/1381612823666170622095324. 28641532

[35] Fergus C, Barnes D, Alqasem MA, Kelly VP. The queuine micronutrient: charting a course from microbe to man. Nutrients. 2015;7:2897–2929. doi:10.3390/nu7042897. 25884661 PMC4425180

[36] Reisser T, Langgut W, Kersten H. The nutrient factor queuine protects HeLa cells from hypoxic stress and improves metabolic adaptation to oxygen availability. Eur J Biochem. 1994;221:979–986. doi:10.1111/j.1432-1033.1994.tb18814.x. 8181481

[37] Langgut W, Kersten H. The deazaguanine-derivative, queuine, affects cell proliferation, protein phosphorylation and the expression of the proto oncogenes c-fos and c-myc in HeLa cells. FEBS Lett. 1990;265:33–36. doi:10.1016/0014-5793(90)80877-L. 2114310

[38] Langgut W, Reisser T, Nishimura S, Kersten H. Modulation of mammalian cell proliferation by a modified tRNA base of bacterial origin. FEBS Lett. 1993;336:137–142. doi:10.1016/0014-5793(93)81627-C. 8262197

[39] Pathak C, Vinayak M. Modulation of lactate dehydrogenase isozymes by modified base queuine. Mol Biol Rep. 2005;32:191–196. doi:10.1007/s11033-004-6941-2. 16172920

[40] Nishimura S. Structure, biosynthesis, and function of queuosine in transfer RNA. Prog Nucleic Acid Res Mol Biol. 1983;28:49–73. 6410456 10.1016/s0079-6603(08)60082-3

[41] Langgut W, Reisser T, Kersten H. Queuine modulates growth of HeLa cells depending on oxygen availability. Biofactors. 1990;2:245–249. 2282142

[42] Caballero VC, Toledo VP, Maturana C, Fisher CR, Payne SM, Salazar JC. Expression of Shigella flexneri gluQ-rs gene is linked to dksA and controlled by a transcriptional terminator. BMC Microbiol. 2012;12:226. doi:10.1186/1471-2180-12-226. 23035718 PMC3542578

[43] Salazar JC, Ambrogelly A, Crain PF, McCloskey JA, Soll D. A truncated aminoacyl-tRNA synthetase modifies RNA. Proc Natl Acad Sci U S A. 2004;101:7536–7541. doi:10.1073/pnas.0401982101. 15096612 PMC419641

[44] Dubois DY, Blaise M, Becker HD, Campanacci V, Keith G, Giege R, Cambillau C, Lapointe J, Kern D. An aminoacyl-tRNA synthetase-like protein encoded by the *Escherichia coli* yadB gene glutamylates specifically tRNA Asp. Proc Natl Acad Sci U S A. 2004;101:7530–7535. doi:10.1073/pnas.0401634101. 15096594 PMC419640

[45] Luo J, Li YN, Wang F, Zhang WM, Geng X. S-adenosylmethionine inhibits the growth of cancer cells by reversing the hypomethylation status of c-myc and H-ras in human gastric cancer and colon cancer. Int J Biol Sci. 2010;6:784–795. doi:10.7150/ijbs.6.784. 21152119 PMC2999854

[46] Ilisso CP, Castellano M, Zappavigna S, Lombardi A, Vitale G, Dicitore A, Cacciapuoti G, Caraglia M, Porcelli M. The methyl donor S-adenosylmethionine potentiates doxorubicin effects on apoptosis of hormone-dependent breast cancer cell lines. Endocrine. 2015;50:212–222. doi:10.1007/s12020-014-0484-7. 25577236

[47] Treangen TJ, Koren S, Sommer DD, Liu B, Astrovskaya I, Ondov B, Darling AE, Phillippy AM, Pop M. MetAMOS: a modular and open source metagenomic assembly and analysis pipeline. Genome Biol. 2013;14:R2. doi:10.1186/gb-2013-14-1-r2. 23320958 PMC4053804

[48] Robertson CE, Harris JK, Wagner BD, Granger D, Browne K, Tatem B, Feazel LM, Park K, Pace NR, Frank DN, et al. Explicet: graphical user interface software for metadata-driven management, analysis and visualization of microbiome data. Bioinformatics. 2013;29:3100–3101. doi:10.1093/bioinformatics/btt526. 24021386 PMC3834795

[49] Abubucker S, Segata N, Goll J, Schubert AM, Izard J, Cantarel BL, Rodriguez-Mueller B, Zucker J, Thiagarajan M, Henrissat B, et al. Metabolic reconstruction for metagenomic data and its application to the human microbiome. PLoS Comput Biol. 2012;8:e1002358. doi:10.1371/journal.pcbi.1002358. 22719234 PMC3374609

[50] Mendez R, Del Carmen Piqueras M, Raskind A, de Jong FA, Beecher C, Bhattacharya SK, Banerjee S. Quantitative metabolomics using isotope residue outlier analysis (IROA((R))) with internal standards. Methods Mol Biol. 2019;1996:41–46. 31127545 10.1007/978-1-4939-9488-5_4

