# Supplementary material for: Obesity enriches for tumor protective microbial metabolites and treatment refractory cells to confer therapy resistance in PDAC
Source: Gut Microbes. 2022 Jul 11;14(1):2096328. doi: 10.1080/19490976.2022.2096328 (PMC9275504; doi:10.1080/19490976.2022.2096328)
Supplement: Supplemental Material [file KGMI_A_2096328_SM3232.pdf]

Supplementary Figure legends

Supplementary Figure 1. Gut microbiome changes in lean vs obese animals :Relative abundance was observed at the class level as well (**A**). There was no observable difference in the relative abundance between the lean and obese animals treated with Gem/Pac (**B)**.

Supplementary Figure 2: Metabolic and physiological changes in obese animals Animals on high fat diet predictably showed increase in body weight after 30 days on their diet, whether adjusted control/lean or high fat/obesogenic (**A**). An increase in blood glucose (**B**), triglyceride (**C**) and cholesterol (**D**) levels was observed in the obese mice on high fat diet compared to those on adjusted control diet. There was no significant difference in the tumor take between the two groups (**E**). In the animals on high fat diet, the tumor progressed faster compared to those in the adjusted control diet (**F**) and showed accumulation of lipids (**G).** Tumor volume in PANC02 cell derived tumors in Adjusted control diet and high fat diet fed mice. * represents significant difference in the results **(H).**

Supplementary Figure 3. Queuosine induced proliferation in pancreatic cancer cells Mia-PACA2(A) and S2VP10 (B). Estimation of SAM in adjusted control diet fed and high fat diet fed mice. * represents significant difference in the results (C)

Supplementary Figure 4. Gene expression changes with queuosine. Treatment of pancreatic cancer cells with queuosine changed expression of genes as seen in oxidative stress PCR array analysis A-C. Efficacy of silencing PRDX1 in pancreatic cancer cells (D). Silencing PRDX1 did not lead to cell death (E).

Supplementary Figure 5. Obesity and stemness: Serum cytokine analysis of lean vs obese animals (A) Quantitation of CD133+ staining near lipid droplets (B). Summary of adipocyte conditioned media induced increase in CD133+ population n=3 (C). Treatment with adipocyte conditioned media as well as preQ increased expression of drug resistance genes (D) Treatment with pre-Q did not change CSC gene expression (E).

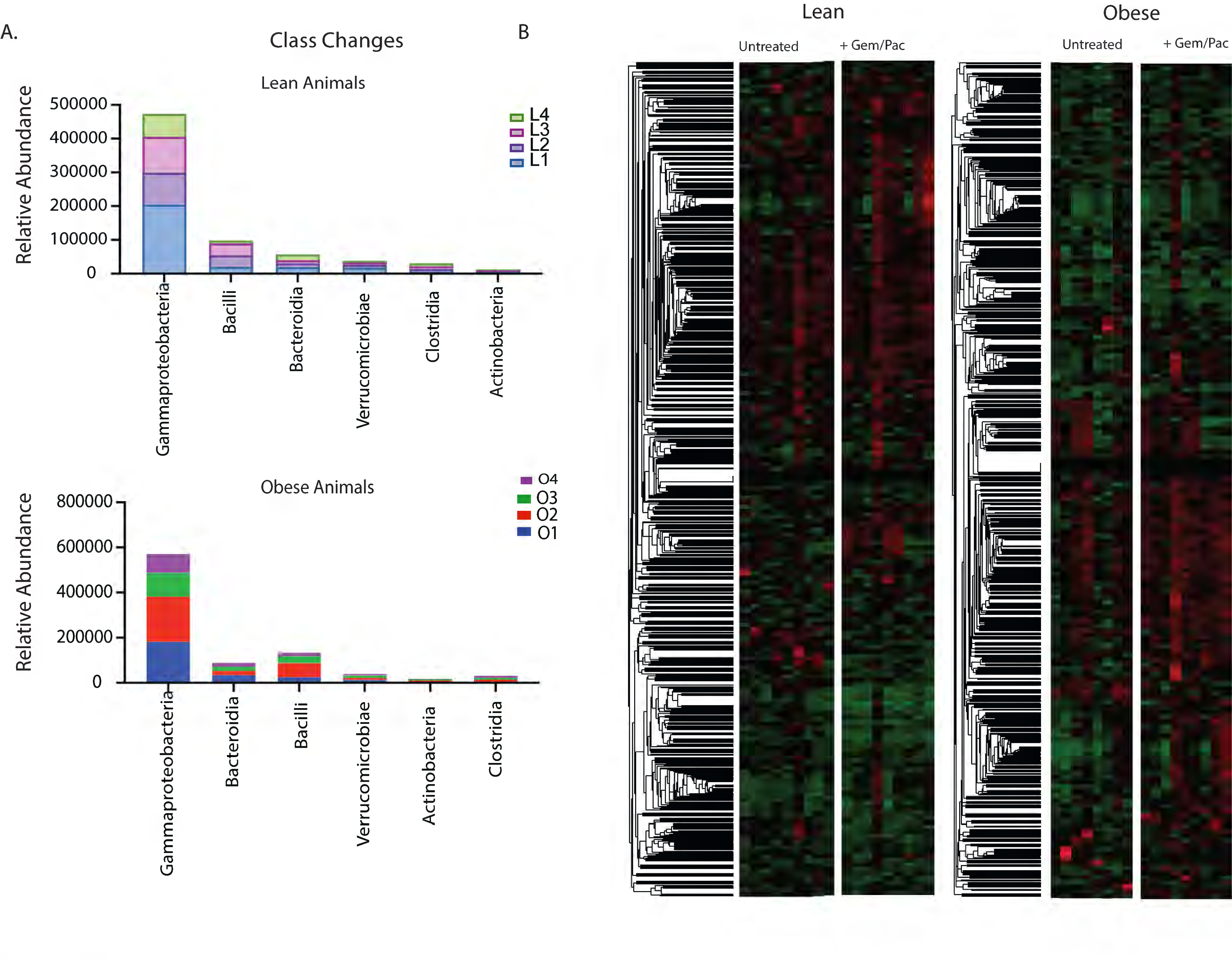

Supplementary Figure 1

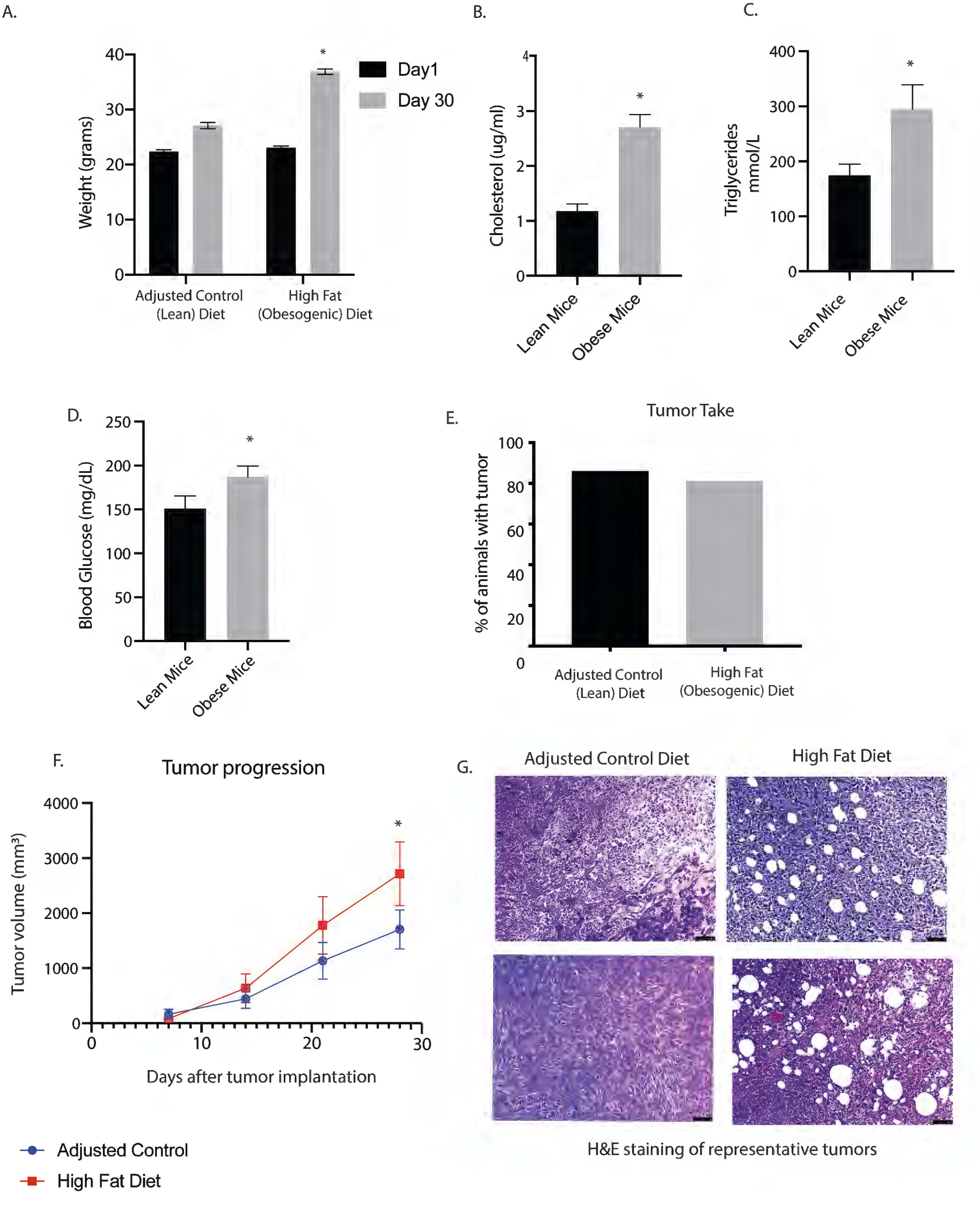

Supplementary Figure 2

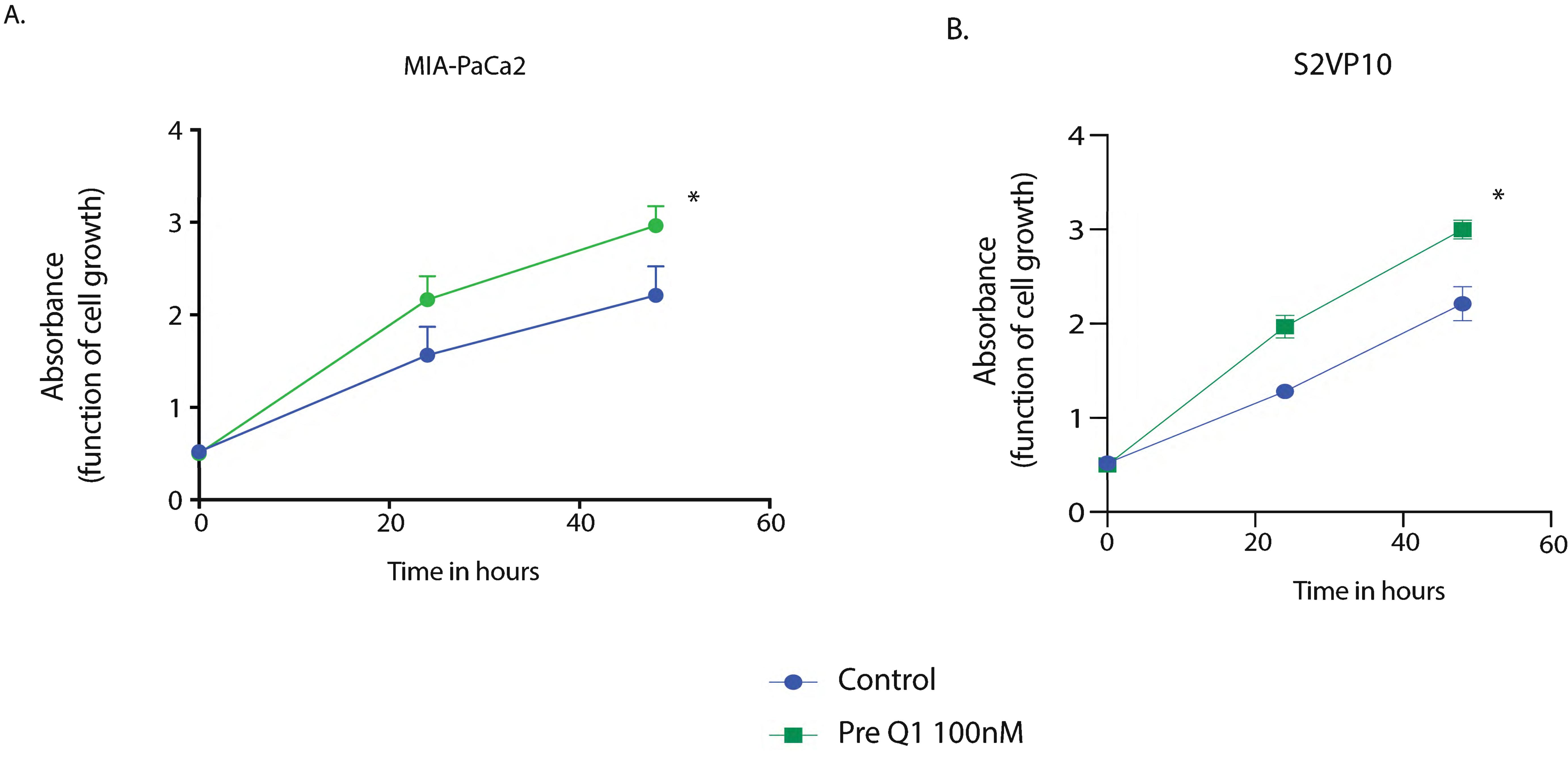

Supplementary Figure 3

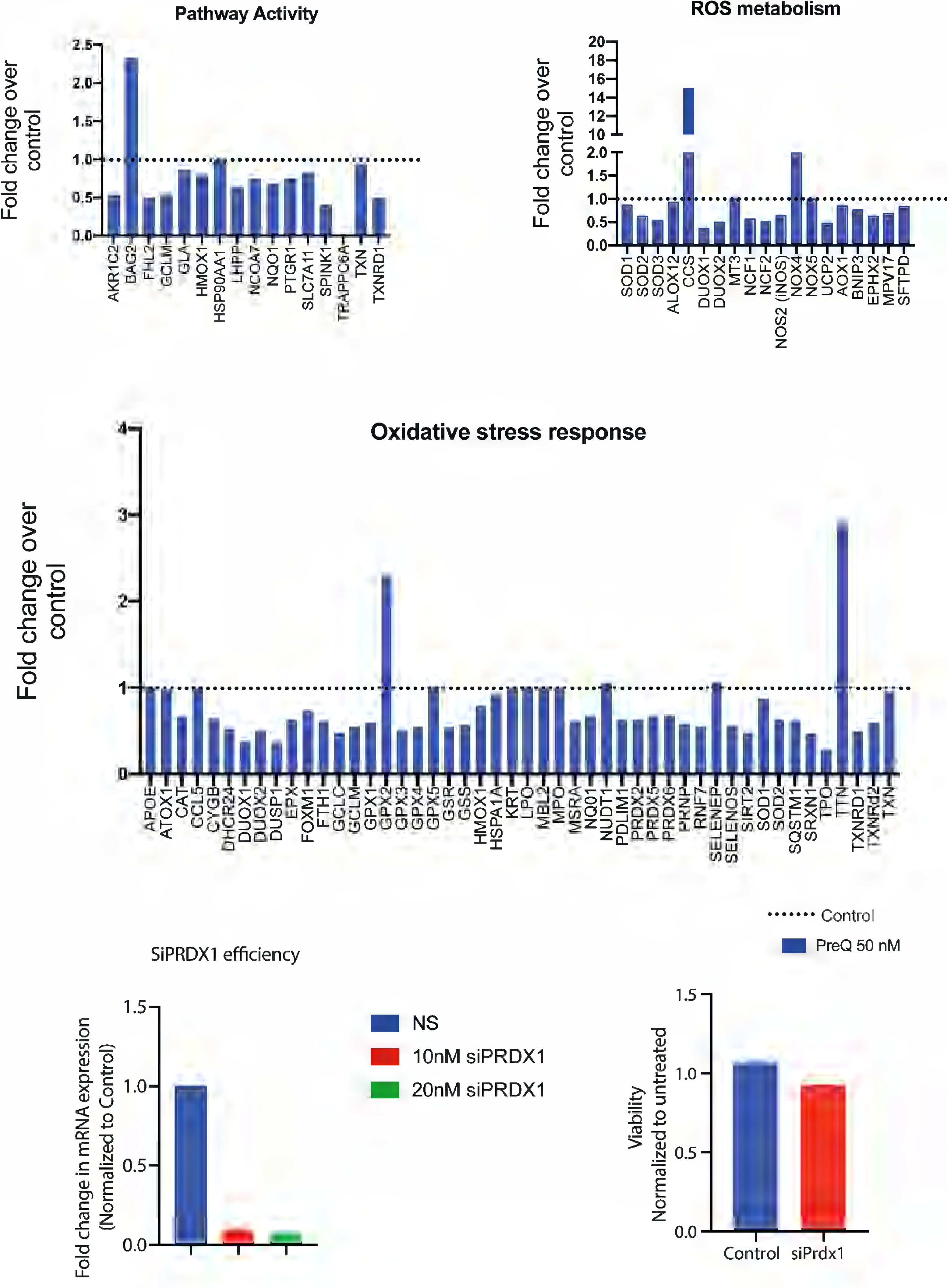

Supplementary Figure 4

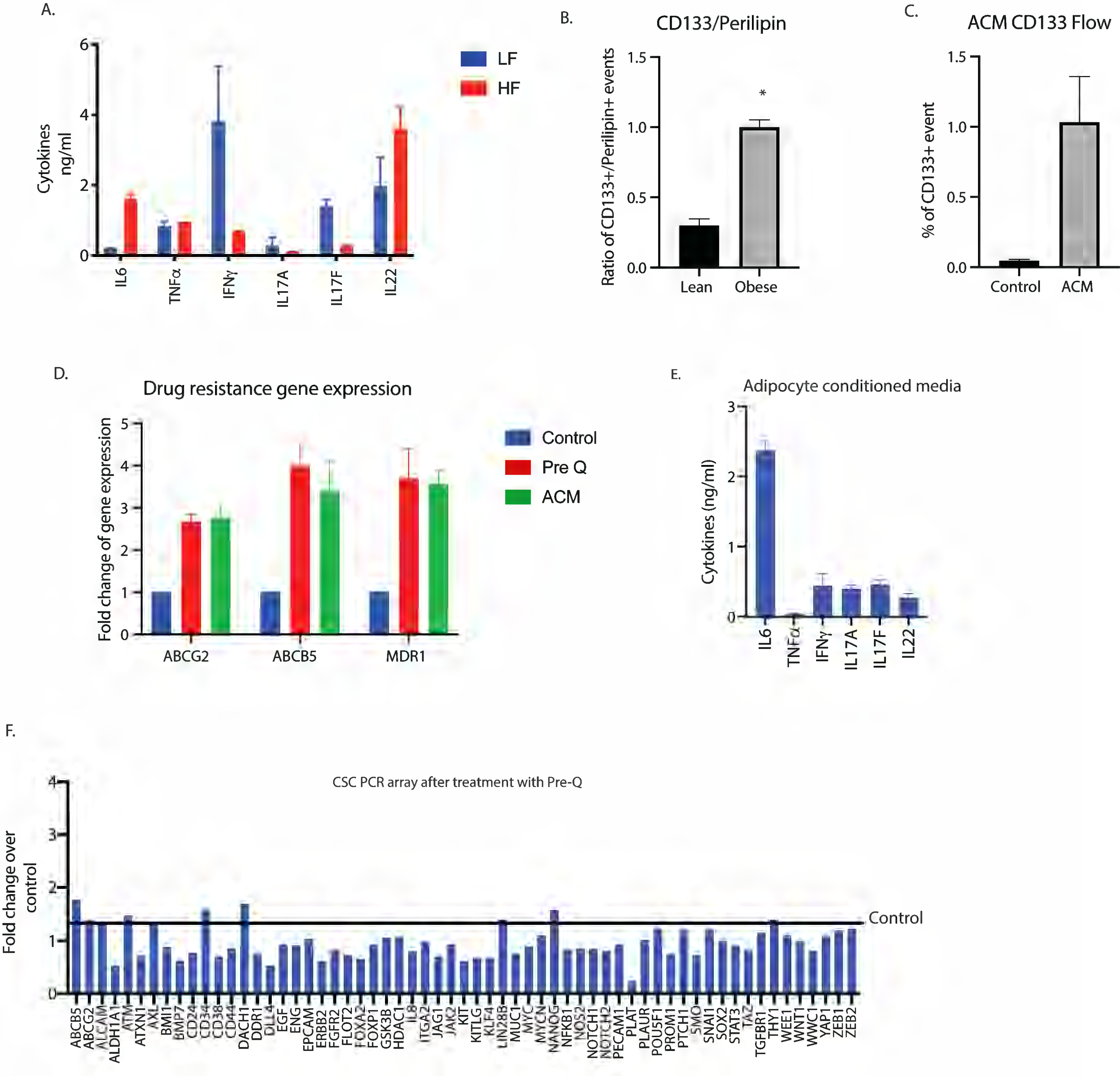

Supplementary Figure 5

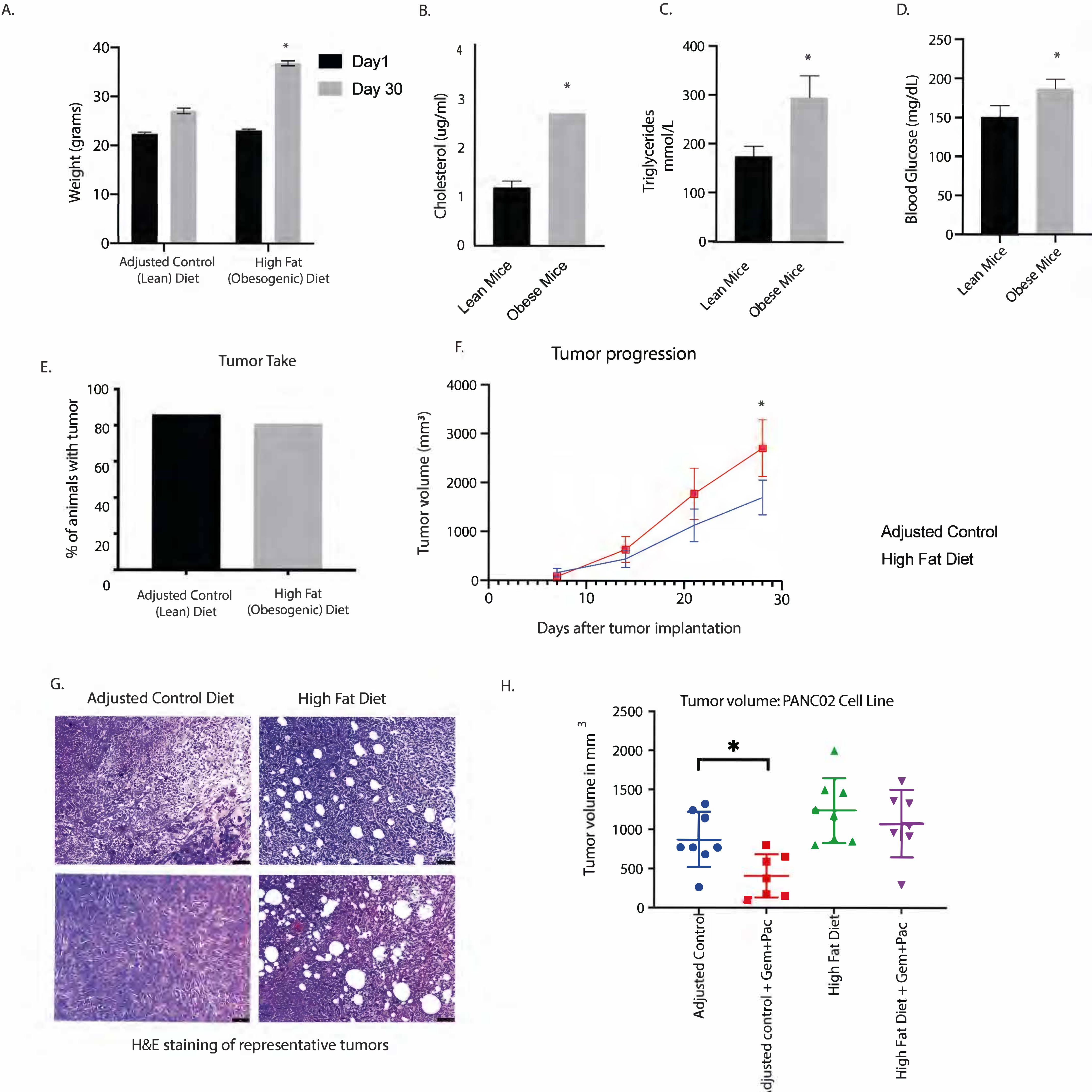

Supplementary Figure 2

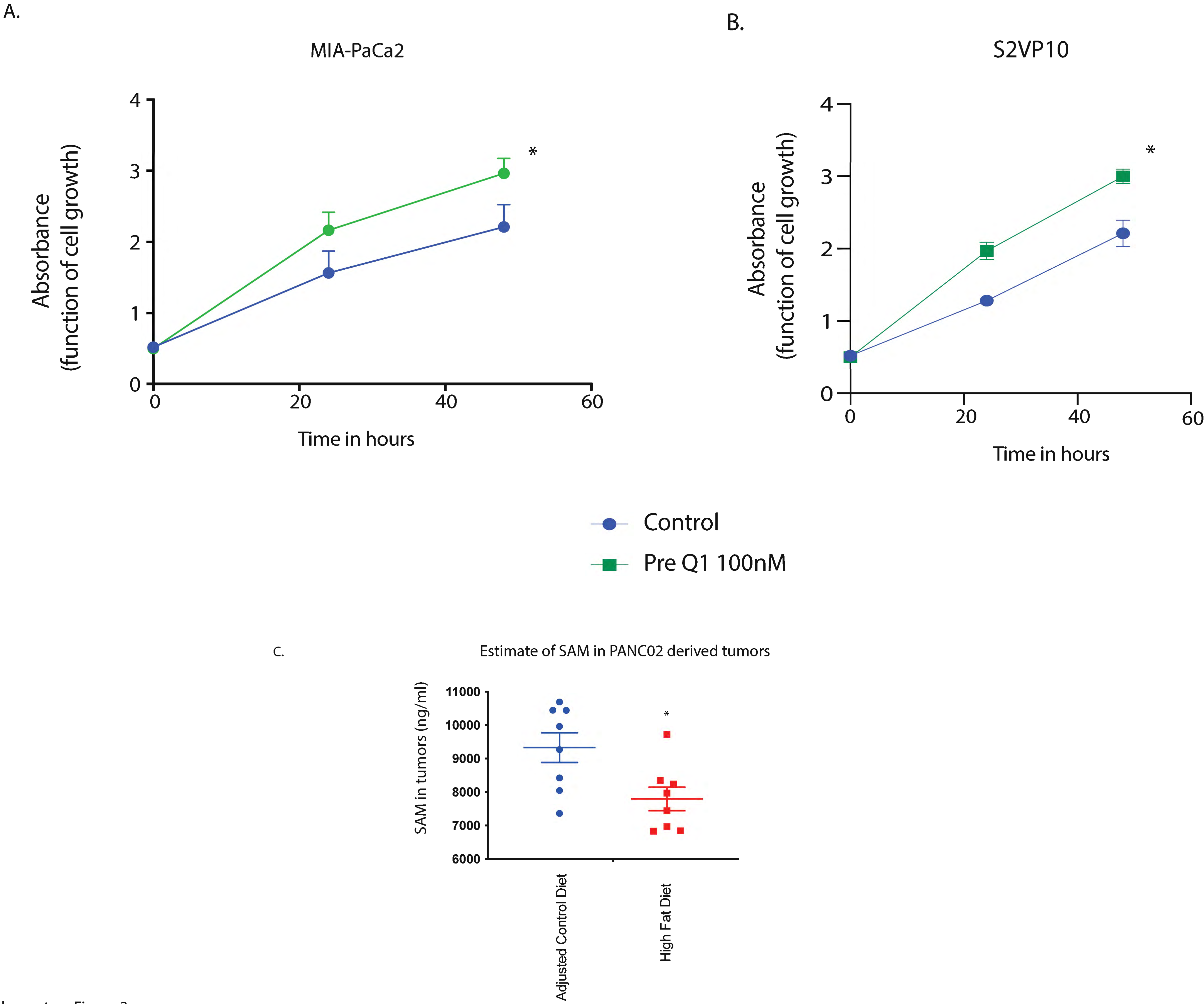

Supplementary Figure 3

Supplementary Figure 4

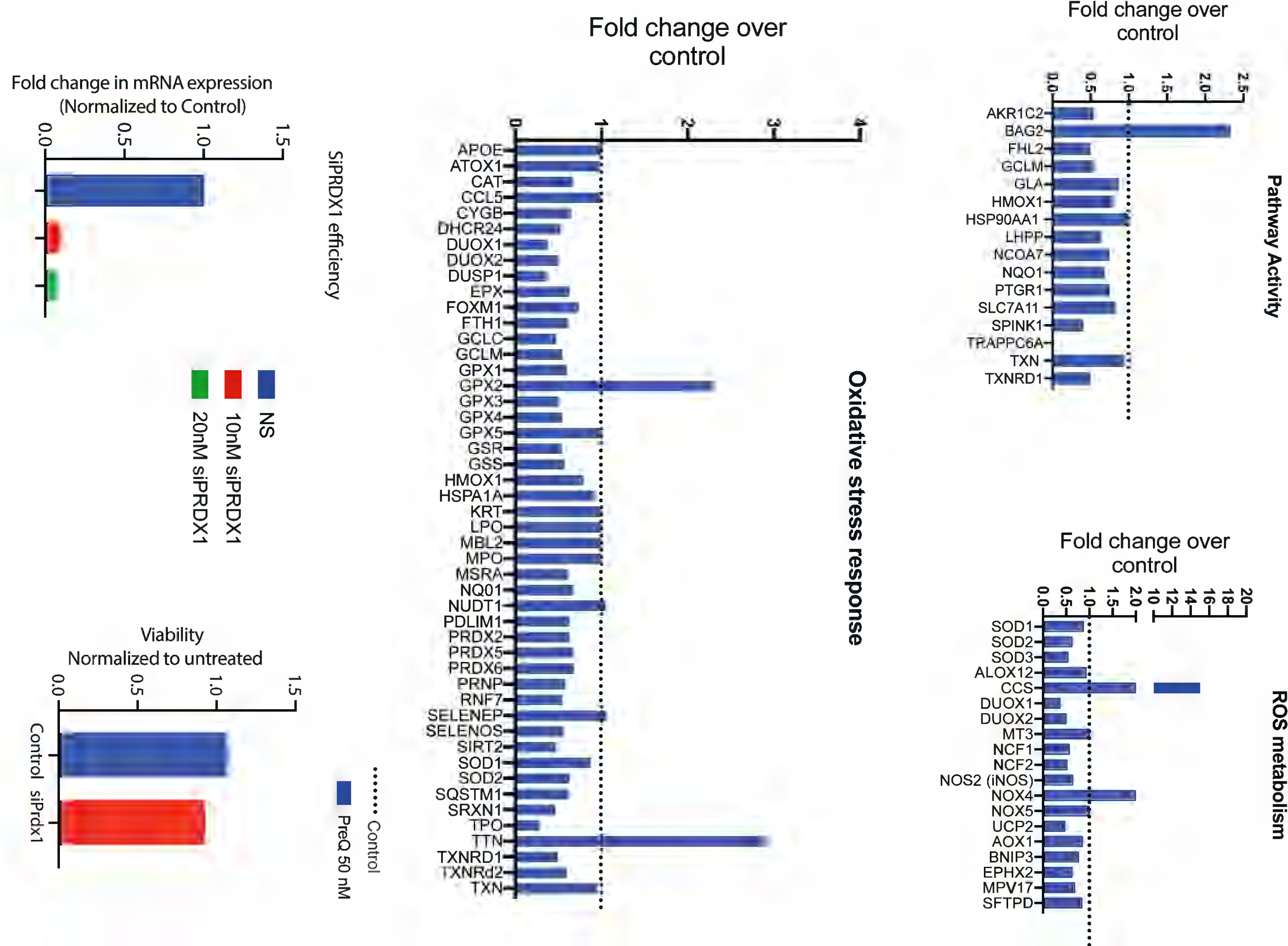

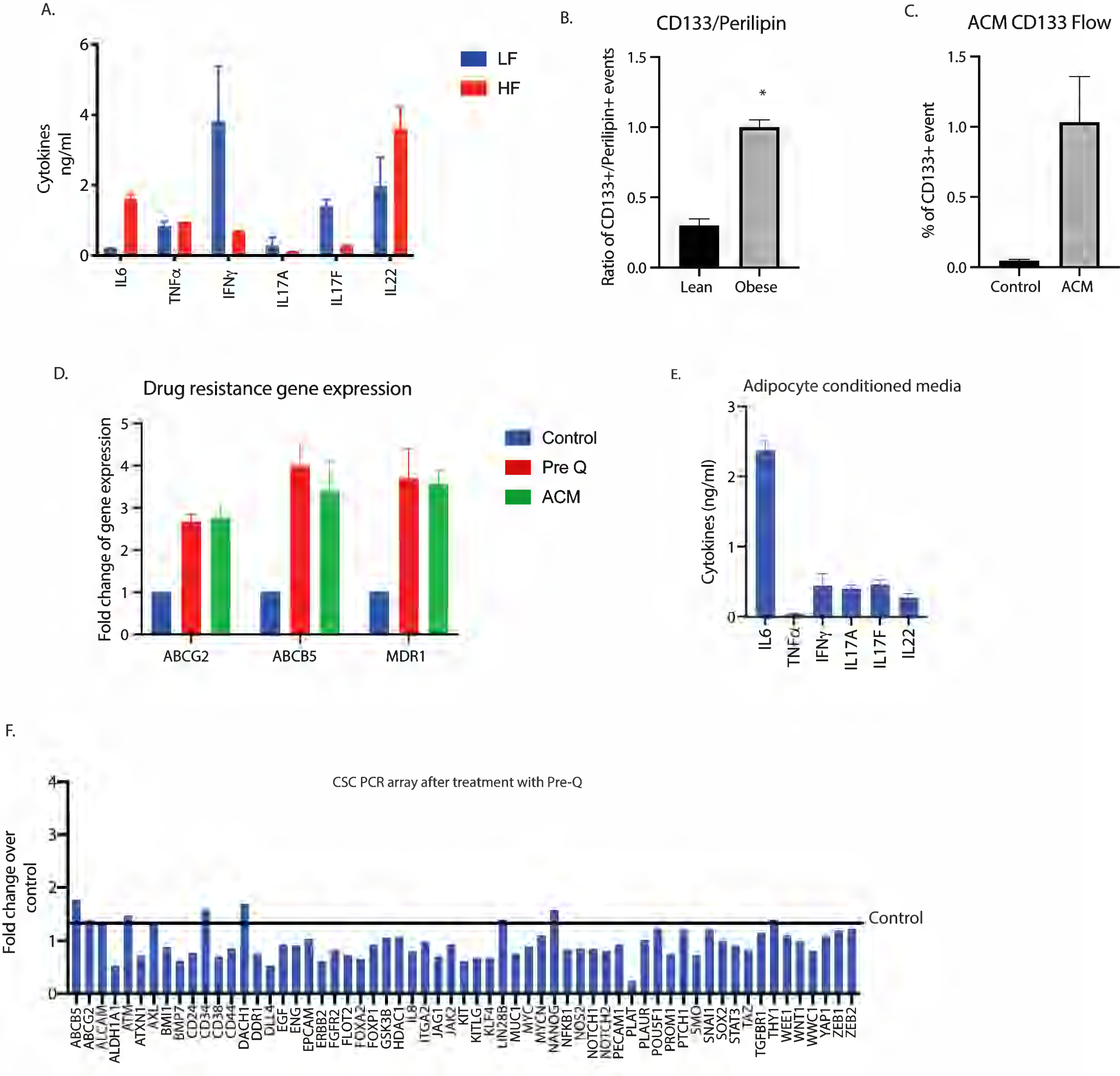

Supplementary Figure 5

metabolite	Sample_1	Sample_2	Sample_3
(AC)2-L-LYS-D-ALA	0	0	0
2-HYDROXY-2-METHYLPROPANENITRILE	0	0	0
2-NAPHTHYLAMINE	0	0	0
ADENOSINE	0	0	0
AMINOIMIDAZOLE RIBOTIDE	0	0	0
AlaAlaProLeu	0	0	0
DOMOILACTONE A	0	0	0
FRUCTOSELYSINE	0	0	0
GLUCOERYSOLIN	0	0	0
GlnLeu	0	0	0
IleGlyArg	0	0	0
L-ALANINE	0	0	0
LEU-GLY-PRO	0	0	0
LeuSerPro	0	0	0
N-BUTYL-N(2)-[(2Z)-2-(2-FURYL)-2-(METHOXYIMINO)ACET	0	0	0
N-HYDROXYDEBRISOQUINE O-GLUCURONIDE	0	0	0
PIPERIDEINE	0	0	0
QUINOLINE YELLOW	0	0	0
SUBAPHYLLIN	0	0	0
SUCROSE	0	0	0
TrpSer	0	0	0
XANTHINE	0	0	0
N-ACETYLNEURAMINATE	0	0.014	0.122
MONO(2-ETHYLHEXYL) PHTHALATE(1-)	0	0.054	0.209
L-METHIONINE S-OXIDE	0.001	0.022	0.039
CARBETAMIDE	0.035	0	0
DIETHYLPHOSPHORIC ACID	0.011	0.011	0.009
ADENINE	0.572	0.917	1.693
ARG-OET	0.696	0.392	0.065
7,8-DIAMINONONANOATE	0	5.328	6.186
BENZOATE	3.071	2.182	1.669
LIBRAMYCIN A	0.565	0.279	0.085
L-THREONINE	0.011	0.011	0.016
L-GLUTAMIC ACID	0.615	169.823	289.421
URIDINE-5-MONOPHOSPHATE	0.025	0.082	0.046
METHOHEXITAL	0	0.2	0.101
L-METHIONINE	0.108	0.291	1.008
3-METHYLENEOXINDOLE	3.229	0.131	1.028
Possibly C9H15NO6	0.023	0.032	0.041
O-SUCCINYL-L-HOMOSERINE	21.251	2.635	0.849
GAMMA-GLUTAMYL-GAMMA-AMINOBUTYRALDEHYDE	255.855	36.884	132.587
L-PHENYLALANINE	9.071	0	0.013
4-AMINOBUTANOATE	0.005	0.005	0.006
(S)-ATPA	0.366	0.015	0.016

TAUROPINE	0.004	0	2.92
L-LEUCINE	7.49	2.816	2.926
L-PROLINE	168.793	30.677	31.69
SUCCINATE	33.508	15.81	19.309
BRUNFELSAMIDINE	4.003	5.169	3.852
(N-TRIS[HYDROXYMETHYL]METHYL-2-AMINOETHANESULF(	4.154	0.632	0.118
N2-(D-1-CARBOXYETHYL)-L-LYSINE	11.77	3.123	632.531
3',5'-CYCLIC GMP	56.53	0	0
GAMMA-L-GLUTAMYL-D-ALANINE	1.605	0.768	0.477
CHLORDANE	1.692	4.354	1.843
6-[(4-HYDROXY-3-NITROPHENYL)ACETAMIDO]CAPROATE	5.31	0	0
1-AMINOCYCLOPROPANE-1-CARBOXYLATE	5.024	5.714	4.426
LL-2,6-DIAMINOHEPTANEDIOATE	22.323	1.369	4.836
5-BENZYL-1-(2-HYDROXYETHOXYMETHYL)URACIL	2.445	15.074	34.989
N(2)-[(R)-2-AMINO-2-PHENYLACETYL]-N-BUTYL-L-SERINAM	0.026	0	0
SN-GLYCERO-3-PHOSPHOETHANOLAMINE	7.856	6.332	1.724
N(6)-ACETONYLLYSINE	7.559	6.885	1.876
L-ORNITHINE	0.04	0.178	0.198
PIPECOLATE	0.968	0.881	0.867
4-OXOPROLINE	19.446	26.152	40.148
L-ALPHA-GLYCEROPHOSPHOCHOLINE	4.339	12.786	4.933
L-TYROSYL-L-ARGININE	0	0	0.469
GANCIDIN W	29.648	1.019	3.182
FRUCTOSELYSINE 6-PHOSPHATE	0.815	0.54	0.436
2,4-DIACETAMIDO-2,4,6-TRIDEOXY-BETA-L-ALTROSE	0.997	1.058	1.24
L-TYROSINE	17.234	26.062	20.728
N-(6-AMINOHEXANOYL)-6-AMINOHEXANOATE	3.376	1.649	5.392
L-VALINE	45.695	5.801	51.649
METHYL-CCNU	4.088	0	1.77
PIPERIDINE	2.592	1.739	1.88
Possibly C4HN3O5P5	1.295	1.102	1.799
N6-ACETYL-N6-HYDROXY-L-LYSINE	37.403	42.004	30.894
BETA-ALANYL-L-LYSINE	16.294	7.881	12.628
INDOLE	31.051	11.506	12.76
N-PHENYLACETAMIDE	1.941	2.023	1.758
L-ASPARTATE	18.426	10.495	6.447
L-GLUTAMINE	14.771	14.139	14.797
PENTALENE	0.013	0.012	0.01
BETA-ALANYL-L-ARGININE	12.036	0	29.958
L-ARGININE	44.623	37.095	42.838
4,6-DIDEOXY-4-(3-DEOXY-L-GLYCERO-TETRONAMIDO)-2-O-	1.018	0.166	0.595
PROCLAVAMINIC ACID	4.424	3.657	5.126
4-GUANIDINOBUTANAL	3.97	3.705	3.892
L-SERINE	0.932	1.539	4.558
L-HISTIDINE	18.536	13.086	13.866

2-(6`-METHYLTHIO)HEXYLMALIC ACID	0.307	6.504	11.51
L-ASPARAGINE	0.677	3.355	0.302
DIACETYL	6.328	6.032	5.919
HIS-LEU	0.368	0.275	0.126
GLYCYL-LEUCINE	118.55	137.731	122.92
L-LYSINE	8.073	1.855	2.757
ADENOSINE 5'-MONOPHOSPHATE	4.427	12.397	0.922
MURAMIC ACID	0.132	0.03	0.053
INDOLINE	1.075	1.004	1.002
UNK MZ 190.1437 RT 0.60	50.777	495.861	316.308
WIN54954	0.016	0	0
L-TRYPTOPHAN	166.953	51.873	0.691
5'-METHYLTHIOADENOSINE	0.506	3.289	0.027
3',5'-CYCLIC AMP	0.099	0	0

Sample_4	Sample_5	LF	Sample_8	Sample_9	Sample_10	Sample_11
0	0	0	0	0	0	0
0	0	0	0	0.178	0	0
0	0	0	0	9.297	25.927	0
0	0	0	54.321	0.019	0	0
0	0	0	0	6.278	0	0
0	0	0	0	0.038	0	0
0	0	0	0	0.116	0	0
0	0	0	0	0	0	0
0	0	0	0	0	0	0
0	0	0	0	0	0	0
0	0	0	0	0	0	0.087
0	0	0	8.469	14.418	0	0
0	0	0	0	0	0	0
0	0	0	0	0.214	0	0
0	0	0	0.087	0.037	0	0
0	0	0	0	0	0	0
0	0	0	0	0.768	0	0
0	0	0	0	0	0	0
0	0	0	13.07	0.459	0	0
0	0	0	0.348	0	0	0
0	0	0	0	0	0	0
0	0	0	0.192	0	0	0
0.112	0.019	0.0534	23.034	0	0.019	0.022
0.357	0	0.124	40.136	0.553	1.323	5.369
0.063	0.009	0.0268	0.374	5.579	0.059	0.054
0.048	0	0.0166	1.763	0.043	0.028	0
0.01	0.008	0.0098	0.06	0.981	0.008	0.01
0.793	0.166	0.8282	33.44	0.461	1.627	17.151
0.34	0.321	0.3628	0.766	16.844	0.176	0.698
2.138	1.451	3.0206	10.568	84.727	12.622	3.376
1.913	2.321	2.2312	77.294	1.265	0.648	1.511
0	0.246	0.235	1.444	0.433	0.192	0.372
0.009	0.03	0.0154	0.024	0.317	0.004	0.119
197.472	48.312	141.1286	1444.951	13.244	104.621	1.029
0	0.054	0.0414	0.182	0.349	0.078	0.058
0.296	0.337	0.1868	0	0.28	1.083	0.851
0.848	0.072	0.4654	0.396	3.311	0.26	1.098
0.354	0.089	0.9662	2.226	8.124	0.182	0.175
0.058	0.061	0.043	0.024	0.261	0.038	0.072
3.537	13.56	8.3664	0.61	69.165	1.4	6.493
112.779	174.674	142.5558	151.929	233.796	522.378	205.532
0.01	0.001	1.819	1.605	8.071	3.922	3.41
0.006	0.006	0.0056	0.005	0.033	0.004	0.005
0.012	0.118	0.1054	0.107	0.322	0.175	0.344

0.308	1.91	1.0284	0	0.046	0.916	6.558
4.985	3.827	4.4088	4.379	10.562	5.947	11.594
70.209	89.271	78.128	56.083	7.572	20.183	95.89
3.139	22.306	18.8144	25.044	44.778	21.303	29.18
4.005	2.95	3.9958	10.137	5.91	3.833	3.597
1.334	1.569	1.5614	3.593	2.997	0.141	4.153
368.938	278.142	258.9008	297.463	7.495	194.364	540.004
0	0	11.306	0	0	0	78.424
0.783	0.411	0.8088	2.701	0.486	0.795	0.923
4.398	3.586	3.1746	0.668	2.125	1.326	7.375
0	0	1.062	6.828	0	0	0
6.043	4.649	5.1712	4.71	0	4.087	5.894
12.263	15.291	11.2164	5.454	10.997	0.926	12.107
5.235	0	11.5486	50.234	2.835	9.256	6.673
0	0.04	0.0132	0.034	0	0.02	0
2.39	2.694	4.1992	4.023	3.256	7.732	3.77
7.646	4.318	5.6568	20.733	0	2.196	6.365
0.183	0.078	0.1354	0.248	0.066	0.175	0.111
0.876	0.91	0.9004	0.726	1.987	0.614	0.882
18.377	36.571	28.1388	40.943	9.312	42.156	26.116
0	12.455	6.9026	7.339	10.883	8.003	1.926
0	0	0.0938	0.508	0	0	0
0.75	14.019	9.7236	4.564	3.299	1.334	1.255
1.121	0.495	0.6814	0.295	0.812	0.676	0.766
2.423	0.669	1.2774	0.494	1.68	1.854	1.23
21.404	21.33	21.3516	16.842	15.316	24.447	22.232
0.243	9.593	4.0506	3.837	0.776	5.974	2.377
40.218	34.078	35.4882	50.683	17.148	30.512	30.93
0	5.772	2.326	5.026	0	0	0
3.058	2.281	2.31	1.983	0.857	1.308	3.448
2.993	1.44	1.7258	0.637	4.183	1.012	1.438
36.416	107.255	50.7944	46.564	12.379	44.735	42.998
9.396	10.711	11.382	17.801	0.032	5.316	17.462
12.491	9.249	15.4114	6.534	7.9	1.655	24.244
3.532	1.219	2.0946	1.223	0.283	1.593	1.842
5.857	24.766	13.1982	14.782	6.492	8.454	11.068
14.276	18.241	15.2448	3.767	18.656	8.808	14.074
0.007	0.007	0.0098	0.01	0.005	0.01	0.005
27.369	0	13.8726	44.202	0	0	0
46.292	61.223	46.4142	14.758	0.105	49.246	60.886
0.277	1.494	0.71	0.378	0	0.832	0.996
4.69	8.039	5.1872	1.106	0.389	3.771	5.86
4.84	4.75	4.2314	4.879	0.038	1.802	3.956
0.029	0.387	1.489	2.021	0.564	0.763	1.655
16.04	22.073	16.7202	5.741	11.444	13.463	13.674

15.595	15.74	9.9312	10.046	2.362	13.607	5.783
2.97	0.602	1.5812	0.032	0.078	2.35	1.616
2.288	2.483	4.61	4.54	0.474	4.173	1.066
0.257	0.412	0.2876	0.113	0	0.278	0.235
261.527	104.114	148.9684	31.494	41.848	94.153	79.439
3.088	2.491	3.6528	1.478	2.827	1.165	1.846
2.187	2.21	4.4286	2.177	0.498	3.411	2.618
0.113	0.075	0.0806	0.018	0	0.03	0.022
0.724	1.867	1.1344	0.663	0.26	0.375	0.425
27.055	15.899	181.18	93.58	28.309	116.153	51.428
0.016	0.018	0.01	0	0	0	0.013
53.19	0	54.5414	44.623	6.772	0	0
2.194	0	1.2032	0.032	0.236	0.109	0.142
0	0	0.0198	0	0	0	0

Sample_12	HF	
0	0	#DIV/0!
0	0.0356	#DIV/0!
0	7.0448	#DIV/0!
0	10.868	#DIV/0!
0	1.2556	#DIV/0!
0	0.0076	#DIV/0!
0	0.0232	#DIV/0!
0	0	#DIV/0!
0	0	#DIV/0!
0	0	#DIV/0!
0	0.0174	#DIV/0!
0	4.5774	#DIV/0!
0	0	#DIV/0!
0	0.0428	#DIV/0!
0	0.0248	#DIV/0!
0	0	#DIV/0!
0	0.1536	#DIV/0!
0	0	#DIV/0!
0	2.7058	#DIV/0!
0	0.0696	#DIV/0!
0	0	#DIV/0!
0	0.0384	#DIV/0!
0.032	4.6214	86.5430712
0	9.4762	76.4209677
0.009	1.215	45.3358209
0	0.3668	22.0963855
0.008	0.2134	21.7755102
0.39	10.6138	12.8155035
0.266	3.75	10.3362734
6.872	23.633	7.82394226
1.952	16.534	7.41036214
5.721	1.6324	6.94638298
0.036	0.1	6.49350649
738.258	460.4206	3.26241882
0	0.1334	3.22222222
0	0.4428	2.37044968
0.115	1.036	2.22604211
0	2.1414	2.21631132
0.078	0.0946	2.2
11.229	17.7794	2.12509562
223.702	267.4674	1.87622952
0.003	3.4022	1.87036833
0.004	0.0102	1.82142857
0.006	0.1908	1.81024668

1.384	1.7808	1.73162194
4.517	7.3998	1.6784159
410.819	118.1094	1.51174227
15.369	27.1348	1.44223573
4.691	5.6336	1.40988037
0.106	2.198	1.40771103
774.314	362.728	1.40103082
0	15.6848	1.38729878
0.607	1.1024	1.36300692
9.171	4.133	1.3018963
0	1.3656	1.28587571
17.038	6.3458	1.22714264
38.776	13.652	1.21714632
0	13.7996	1.1949154
0.024	0.0156	1.18181818
5.492	4.8546	1.15607735
2.849	6.4286	1.13643756
0.159	0.1518	1.1211226
0.796	1.001	1.11172812
34.211	30.5476	1.08560422
9.301	7.4904	1.08515632
0	0.1016	1.08315565
40.068	10.104	1.03912131
0.942	0.6982	1.02465512
0.743	1.2002	0.93956474
21.123	19.992	0.93632327
5.869	3.7666	0.92988693
31.88	32.2306	0.90820611
5.357	2.0766	0.8927773
2.643	2.0478	0.88649351
0.352	1.5244	0.8833005
76.139	44.563	0.87732112
9.263	9.9748	0.87636619
26.434	13.3534	0.86646249
3.799	1.748	0.83452688
12.06	10.5712	0.80095771
12.532	11.5674	0.75877676
0.007	0.0074	0.75510204
7.943	10.429	0.75176968
46.68	34.335	0.73975206
0.293	0.4998	0.70394366
6.734	3.572	0.68861814
3.694	2.8738	0.67916056
0.025	1.0056	0.67535259
11.632	11.1908	0.66929821

0	6.3596	0.64036572
0.394	0.894	0.56539337
2.264	2.5034	0.54303688
0.149	0.155	0.53894298
131.78	75.7428	0.50844877
1.674	1.798	0.49222514
1.328	2.0064	0.45305514
0.089	0.0318	0.39454094
0.494	0.4434	0.39086742
62.561	70.4062	0.38859808
0	0.0026	0.26
0	10.279	0.18846234
0	0.1038	0.08626995
0	0	0
